# HNF1B Alters an Evolutionarily Conserved Nephrogenic Program of Target Genes

**DOI:** 10.1681/ASN.2022010076

**Published:** 2022-12-15

**Authors:** Kelli Grand, Martine Stoltz, Ludovica Rizzo, Ruth Röck, Michael M. Kaminski, Gabriela Salinas, Maike Getwan, Thomas Naert, Roman Pichler, Soeren S. Lienkamp

**Affiliations:** 1 Institute of Anatomy, University of Zurich, Zurich, Switzerland; 2 The University Medical Center Freiburg, Faculty of Medicine, University of Freiburg, Freiburg, Germany; 3 Berlin Institute for Medical Systems Biology, Max Delbrück Center for Molecular Medicine in the Helmholtz Association, Berlin, Germany; 4 Department of Nephrology and Medical Intensive Care, Charité Universitätsmedizin Berlin, Berlin, Germany; 5 Berlin Institute of Health, Berlin, Germany; 6 Transcriptome and Genome Analysis Laboratory, University Medical Center Göttingen, Göttingen, Germany

**Keywords:** HNF1B, genetic renal disease, Xenopus, direct reprogramming, kidney development, transcription regulation

## Abstract

**Significance Statement:**

Mutations in hepatocyte nuclear factor-1 β (*HNF1B*) are the most common monogenic causes of congenital renal malformations. HNF1B is necessary to directly reprogram fibroblasts to induced renal tubule epithelial cells (iRECs) and, as we demonstrate, can induce ectopic pronephric tissue in *Xenopus* ectodermal organoids. Using these two systems, we analyzed the effect of *HNF1B* mutations found in patients with cystic dysplastic kidney disease. We found cross-species conserved targets of HNF1B, identified transcripts that are differentially regulated by the patient-specific mutant protein, and functionally validated novel HNF1B targets *in vivo*. These results highlight evolutionarily conserved transcriptional mechanisms and provide insights into the genetic circuitry of nephrogenesis.

**Background:**

Hepatocyte nuclear factor-1 β (HNF1B) is an essential transcription factor during embryogenesis. Mutations in *HNF1B* are the most common monogenic causes of congenital cystic dysplastic renal malformations. The direct functional consequences of mutations in *HNF1B* on its transcriptional activity are unknown.

**Methods:**

Direct reprogramming of mouse fibroblasts to induced renal tubular epithelial cells was conducted both with wild-type *HNF1B* and with patient mutations. *HNF1B* was expressed in *Xenopus* ectodermal explants. Transcriptomic analysis by bulk RNA-Seq identified conserved targets with differentially regulated expression by the wild-type or R295C mutant. CRISPR/Cas9 genome editing in *Xenopus* embryos evaluated transcriptional targets *in vivo*.

**Results:**

HNF1B is essential for reprogramming mouse fibroblasts to induced renal tubular epithelial cells and induces development of ectopic renal organoids from pluripotent *Xenopus* cells. The mutation R295C retains reprogramming and inductive capacity but alters the expression of specific sets of downstream target genes instead of diminishing overall transcriptional activity of HNF1B. Surprisingly, targets associated with polycystic kidney disease were less affected than genes affected in congenital renal anomalies. Cross-species–conserved transcriptional targets were dysregulated in *hnf1b* CRISPR-depleted *Xenopus* embryos, confirming their dependence on *hnf1b*.

**Conclusions:**

HNF1B activates an evolutionarily conserved program of target genes that disease-causing mutations selectively disrupt. These findings provide insights into the renal transcriptional network that controls nephrogenesis.

## Graphical abstract

**Figure:**
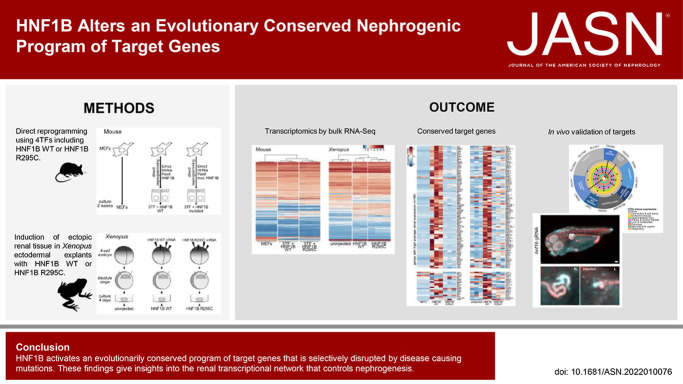

Congenital anomalies of the kidney and urinary tract (CAKUT) represent 20%–30% of prenatal anomalies and are the most common malformation diagnosed in newborns.^1–3^ CAKUT is also associated with 50% of the end-stage renal failure cases in children.^4,5^ Most causal mutations are found in transcription factors involved in renal development. Approximately 30% of pediatric patients have genetic variants in the *HNF1B* gene, making it the most frequently affected locus.^6–8^ Furthermore, pathogenic mutations in *HNF1B* represent the most common single-gene mutations found in congenital kidney disease.^9,10^ *HNF1B* mutations can result in renal cystic dysplasia or other structural abnormalities of the kidney or urinary tract (hypoplasia, electrolyte abnormalities).^6,11–13^ Heterozygous mutations of *HNF1B* are also responsible for maturity-onset diabetes of the young type 5, dysfunction of the liver, and pancreatic atrophy.^14–17^ Diseases associated with *HNF1B* are largely inherited in an autosomal dominant manner, and around 50% of mutations are reported as spontaneous.^8,18^ Patients with heterozygous gene deletions and those with missense mutations can exhibit the same renal phenotype,^18^ suggesting that a loss of gene copy number results in haploinsufficiency. Missense mutations may constitute full or partial loss-of-function alleles. Consistent with these models, homozygous germ line inactivation of *Hnf1b* is embryonically lethal in mice and humans,^18–20^ suggesting that residual protein function is present in patients with *HNF1B*-associated disease.

Hepatocyte nuclear factor-1β (HNF1B) is a member of the homeodomain containing a superfamily of transcription factors and consists of an aminoterminal dimerization domain, a bipartite DNA-binding (POU) domain, and a carboxyterminal transactivation domain.^21,22^ HNF1B binds to DNA by recognizing the consensus sequence as a homo- or heterodimer together with its paralogue HNF1A and interacts with different coactivators and corepressors, including PCBD1.^21–24^ HNF1B can not only activate but also repress the expression of its target genes.^25–28^ The crystal structure of the DNA-binding domain is available (PDB ID: 2H8R) and allows detailed modeling of pathogenic mutations in the POU domains.^29^

Missense mutations are enriched in the DNA-binding domain,^29^ some of which are expected to perturb the DNA-binding properties of the protein. However, different amino acid substitutions at the same position can have vastly different outcomes. For example, substitution of arginine at position 295 with proline (R295P) impaired DNA-binding ability, whereas it was largely retained by a substitution to histidine (R295H).^29^ The R295H mutation can lead to a loss of activity on some Hnf1b target genes *in vitro* while not affecting others. However, whether such gene-specific transcriptional activity is common with other Hnf1b mutations or relevant *in vivo* has not been determined.^22^ Thus, the effect of specific mutations is hard to predict and may range from weakening transcriptional activity across all HNF1B target genes to affecting only distinct sets of target genes more specifically. Hence, the functional effects of individual mutations on the transcriptome remain elusive.

HNF1B can be detected already at early stages of embryonic development and is involved in the tissue-specific regulation of gene expression and the embryonic development of various organs including the liver, kidney, intestine, pancreas, and genitourinary system.^23,30^ It is required at several stages of kidney development, including ureteric bud branching, nephron patterning, and tubulogenesis. The absence of Hnf1b in early kidney development causes defective ureteric bud branching and the lack of mesenchymal to epithelial transition.^8^ If *Hnf1b* is deleted when tubules have already formed but are still elongating, a polycystic phenotype is observed.^31^ However, when *Hnf1b* is deleted in the metanephric mesenchyme alone, nephron precursors are able to form glomerular structures, but tubular expansion and differentiation is completely impaired, resulting in glomerular cysts commonly observed in human patients.^32^ Modeling Hnf1b-related disease in mice has proven challenging. Heterozygous deletions of the gene have no obvious phenotypic consequences.^23^ Recently, a novel mouse was generated by introducing a splice donor site mutation, leading subsequently to reduced Hnf1b protein levels in heterozygous mice and recapitulating much of the human renal pathology.^33^ Currently, no model exists to determine the effect of pathogenic missense mutations of HNF1B on its transcriptional activity on a gene-by-gene basis.

HNF1B is expressed along all nephron segments in the mature kidney. It plays an essential role in nephrogenesis and is also involved in tissue maintenance and injury response of renal tubules.^34,35^ In postnatal kidneys, HNF1B controls genes responsible for metabolism and solute transport and mutations in *HNF1B* can cause a disturbance in electrolyte balance.^36,37^ For example, HNF1B has a physiologic function in renal magnesium handling by regulating the expression of *FXYD2*.^38^ In most cases of HNF1B-associated CAKUT, early nephrogenesis seems to occur normally and allows a functional kidney to be formed initially. Which processes are specifically disrupted by *HNF1B* mutations at later stages, however, remains unknown.

Hnf1b is one of four transcription factors that can directly convert fibroblasts into renal tubule-like epithelial cells (iRECs).^39^ Together with Pax8, Hnf1b is essential for this type of direct reprogramming, suggesting a pivotal role for Hnf1b in defining renal tubular cell identity, not only *in vivo* but also in a “*de novo*” generated cellular model. iRECs have similar functional, transcriptional, and morphologic characteristics to primary renal tubule cells. However, in contrast to primary cells, reprogrammed iRECs retain a high degree of differentiation after cell sorting and long-term passaging. Thus, they offer an attractive model to characterize the nephrogenic potential of individual genes and have been used to characterize the effect of patient-specific mutations in *HNF4A*.^40^

Similarly, ectodermal explants of pregastrula-stage *Xenopus* embryos (animal caps) retain pluripotency and can differentiate into renal tubular tissue on treatment with BMP ligands (activin A) and retinoic acid.^41^ Thus, these *ex vivo* organoids are a suitable tool to characterize key players of renal organogenesis. In addition, *Xenopus* is an established model to explore evolutionarily conserved mechanisms of renal tubulogenesis *in vivo*.^42–44^ Many early inductive factors that specify renal fate retain a similar function to their mammalian orthologs, but also the patterning of the segmental architecture and differentiation of renal tubules in tadpoles mimics that of mature mammalian nephrons.^45,46^ In *Xenopus*, the expression of *hnf1b* begins during specification of the pronephric field and is maintained throughout differentiation with highest expression in the proximal segment.^31^ Some patient-specific mutations of *HNF1B* have also been evaluated in this model but relied on transient or transgenic overexpression.^31,34^

In this study, we explore conserved transcriptional programs in renal tubulogenesis and congenital renal malformations by comparative analysis in *Xenopus* and directly reprogrammed mammalian cells. We find that three transcription factors (Hnf1a, Hnf1b, Sall1) can induce renal tubular tissue in the absence of other external factors when expressed in *Xenopus* ectodermal explants. Focusing on Hnf1b, we identify a patient-specific mutation (R295C) associated with cystic dysplastic kidneys that retains the ability to both induce renal tissue in *Xenopus* explants and convert fibroblasts into induced renal cells in conjunction with three other factors. Comparing the differentially expressed genes of the cell-type conversion events in both species, we identify conserved transcriptional programs activated by HNF1B and find novel target genes. This includes not only known Hnf1b-regulated genes, such as Mg^2+^ transporters, and components of the proximal tubule endocytic uptake machinery but also previously unknown Hnf1b targets associated with cystic kidney disease and renal malformation. Thus, the combined use of directly reprogrammed mammalian cells and *Xenopus* organoids allowed us to gain a unique perspective into evolutionary conserved mechanisms of renal development that may be disrupted in human disease.

## Materials and Methods

### Molecular Cloning

The HNF1B transcript NM_000458.4 (coding isoform 1; NP_000449.1) was cloned into pWPXLd containing an F9 tag. pWPXLd was a gift from Didier Trono (Addgene plasmid # 12258; RRID:Addgene_12258). Mutations Arg165Pro, Gln182X, and Arg295Cys were introduced using the Q5 Site-directed Mutagenesis Kit (NEB, E0554S). Mutations Pro159Leu, Gln253Pro, Pro265Ser, Gly285Asp, and Trp299Gly were cloned using PfuTurbo DNA Polymerase (Agilent Technologies, 600255). The primers were designed using PrimerX (https://www.bioinformatics.org/primerx/) and NEBase Changer (https://nebasechanger.neb.com/) (Supplemental Table 6). The correct sequence of all plasmids was confirmed by Sanger sequencing.

#### *In Vitro* mRNA Transcription

HNF1B WT, R295C transcripts, and cDNA encoding the 13 transcription factors used in the animal cap expression screen were cloned into the VF10 plasmid.^47^ Linearized vectors were purified with phenol-chloroform cleanup, and mRNA was transcribed using mMESSAGE mMACHINE T7 Transcription Kit T7 (Thermo Fisher, AM1344). RNA was cleaned up with RNeasy Kit (Qiagen, 74104). Primers designed for cloning are presented in Supplemental Table 7.

### Cell Culture

#### Reprogramming

All the methods were conducted as described in Kaminski *et al*.^39^ Shortly, lentiviruses containing patient-specific mutations were produced using HEK 293T/17 cells (ATCC, CRL-11268). The pWPXLd vector containing HNF1B WT or mutated gene together with vectors psPAX2 (Addgene, plasmid #12260) and pMD2.G (Addgene, plasmid #12259) were transfected using the calcium phosphate method. After two days, viruses were harvested. For reprogramming, Ksp-Cre reporter MEFs (Cdh16-Cre) were obtained from limbs of E13.5 mouse embryos and kept in a MEF medium (MEFM) containing DMEM, 10% fetal bovine serum (FBS; Sigma-Aldrich, F9665), 1% L-glutamine (Life Technologies, 25030024), and 1% penicillin/streptomycin (Life Technologies, 15140-122). After reaching confluency, cells were split 1:4 and transduced lentivirally with the transcription factors Pax8, Emx2, Hnf4a, and HNF1B WT or mutated HNF1B. Lentiviruses were diluted 1:100 to 1:800 in MEFM containing 8-*μ*g mL^−1^ polybrene (Santa Cruz Biotechnology, sc-134220) and transduced for an average of 17 hours on 7 consecutive days. Reprogrammed, GFP-positive cells were sorted 21 days after the last viral transduction using a BD FACSAria Fusion flow cytometer (Becton Dickinson).

#### 3D Cultures

Trypsinized iRECs were filtered through a 50-*µ*m strainer and resuspended in 6 mg ml^−1^ Matrigel (Matrigel growth factor reduced, Corning, REF 354230) for a final concentration of 1.5·10^5^ cells ml^−1^ and a volume of 40 *µ*l per sample (*i.e.*, 6000 cells per sample). Replicates were dispensed in separated wells of a *µ*-slide 8-well dish (ibidi, Cat. No. 80826) and incubated at 37°C for 20 minutes before addition of a renal epithelial growth medium (Lonza, REGM, CC-3190). 3D embedded cells were cultured for up to 10 days.

#### EMSA

293T/17 ATCC cells (ATCC-CRL-11268, Lot 70022180) were transiently transfected with flag-tagged HNF1B WT, HNF1B R295C, and RFP constructs using a Lipofectamine 3000 reagent (Thermo Fisher Scientific, L3000001). Clarified cell lysates were generated 48 hours after transfection by washing cells in PBS and homogenizing them using a syringe with ten strikes in lysis buffer (20 mM Tris pH 7.5, 1% Triton X-100, protease inhibitor cocktail [Thermo Fisher Scientific, 78429]). Lysates were clarified by centrifugation for 30 minutes at 15,000*g* at 4°C. HNF1B and HNF1B R295C proteins were precipitated by incubating lysates with Anti-DYKDDDDK magnetic agarose (Thermo Fisher Scientific, A36797) for 3 hours at 4°C. Resin-associated proteins were washed three times with lysis buffer and eluted with DYKDDDDK peptide (Thermo Fisher Scientific, A36805). Hybridized 5′ Cy7 labeled oligos (fwd: 5′-CTT GGT TAA TAA TTC ACC AGC-3′, rev: 3′-AA CCA ATT ATT AAG TGG TCG G-5′) were incubated with IP samples HNF1B, HNF1B R295C, and RFP in EMSA-binding buffer (10 mM Tris-HCl pH 7.5, 50 mM NaCl, 1mM MgCl2, 0.5 mM DTT, 0.5 mM EDTA, 100 ug/ml BSA, 4% glycerol) for 30 minutes at RT. EMSA samples were loaded on a 6% TBE polyacrylamide gel and run for 20 minutes at 0.2 mA. DNA bound proteins were visualized using the LI-COR Odyssey DLx machine.

#### Cycloheximide Chase Assay

HEK293 cells were transiently transfected with flag-tagged HNF1B WT, HNF1B R295C, and RFP constructs using the Lipofectamine 3000 transfection reagent (Thermo Fisher Scientific, L3000001). Twenty-four hours after transfection synthesis of new proteins was stopped by treating cells with 300 *μ*g/ul of cycloheximide (Sigma Aldrich, C4859) for 0, 3, 6, 9, 22, 32 hours. Whole-cell lysates were prepared by washing cells 3× with PBS, followed by 10min of ice incubation in lysis buffer (20mM Tris pH 7.5, 1% Triton X-100, protease inhibitor cocktail [Thermo Fisher Scientific, 78429]). 4× Laemmli sample buffer (Bio-rad, 1610747) was added, and samples were heated to 95°C for 5 minutes, followed by a cool down to RT. Cell lysates were then loaded on 10% SDS polyacrylamide gels, followed by Western blotting on a PVDF membrane (Roth, T830.1). Blots were stained with Ponceau S (Carl Roth, 5938.1) and blocked in 2.5% BSA for 1 hour at RT. Incubation with the primary antibody was performed either o/n at 4°C or for 3 hour at RT using anti-FLAG (Sigma Aldrich, F3165) or anti-α-tubulin (DSHB, 12G10), followed by 1h incubation with secondary antibody anti-HRP (Sigma Aldrich, A8924). The ECL detection system (Thermo Fisher Scientific, 32209) was used to visualize proteins using the Vilber Fusion FX machine.

#### Western Blot Analysis

Protein lysates were isolated from transfected HEK 293T/17 cells (ATCC, CRL--11268) and animal cap explants 1 day after the transfection and microinjection experiments. Transfected HEK 293T cells and animal cap explants were homogenized in lysis buffer (1% Triton-X 100, 20 mM Tris pH 7.5, 50 mM NaCl, 50 mM NaF, 15 mM Na4P2O7, 0.1 mM EDTA pH 8.0). For the explants, NaF 10 mM; Na_3_VO_4_, 10 mM; PMSF; and cOmplete (Roche diagnostics) were added. After centrifugation, the supernatant was mixed with the sample buffer (Laemmli Buffer).

Proteins were separated by SDSpolyacrylamide gel electrophoresis, transferred to a PVDF membrane and incubated overnight at 4°C with primary antibodies anti-Flag M2 (Sigma-Aldrich, F3165) and anti-TCF2/VHNF1 (Everest Biotech, EB07588) and 1 hour with goat anti-mouse Ig (Agilent, P0447) and donkey anti-goat IgG-HRP (Santa Cruz Biotechnology, sc-2020) as secondary antibodies for lysates from a 293T cell and *Xenopus* explant, respectively. Chemiluminescence was determined using the ECL detection system ECL. The ECL solution contained TRIS pH 8.5 100 mM (ROTH, 5429.3), 1.5% H_2_O_2_ (Merck, 1.07210.0250) Coumaric Acid 0.4 mM (Sigma-Aldrich, C-9008) Luminol 2.5 mM (Sigma-Aldrich, A8511). We used the X-ray film Super RX (FujiFilm, 4741019284). Mouse anti–γ-tubulin (Sigma-Aldrich, T6557) was used as a loading control (Supplemental Figure 1A).

### Animal Experiments

All experiments on *Xenopus* were conducted as described by Lienkamp *et al.*^48^ and in accordance with local laws and institutional regulations and approved by the regulatory authorities (Regierungspräsidium Baden-Württemberg, Kantonales Veterinäramt Zürich).

#### Xenopus Injections (Knockout and Rescue Experiments)

Natural mating was used to obtain *X. tropicalis* embryos. Mating couples were injected with human chorionic gonadotropin (MSD, Chorulon) 12–72 hours before (10–15U) and on the day of embryo collection (150U). Embryos were transferred into 3% Ficoll dissolved in 0.1× MMR at the four-cell stage and injected with 5 nl into the ventral, vegetal blastomere. For knockout experiments, 0.175 ng of hnf1b and sord sgRNA, respectively, and 0.600 ng of Cas9 protein (PNABio, CP01) were injected. For rescue experiments, 0.175 ng of hnf1b sgRNA and 0.600 ng of Cas9 protein together with 0.005 ng of HNF1B WT or R295C mRNA were injected. Coinjections with 2 ng of fluorescein-dextran (Invitrogen, D1823) were used as a lineage tracer. The sequences for sgRNAs and MOs are presented in Supplemental Table 8. For further analysis, all injected embryos were fixed overnight at 4°C in 1X MEMFA (100 mM MOPS [pH 7.4], 2 mM EGTA, 1 mM MgSO_4_ and 3.7% formaldehyde [Fisher Bioreagents, BP531-500]) in H_2_O, washed and stored in 100% ethanol. Embryo staging was conducted as described by Nieuwkoop and Faber.^49^

#### Genotyping, sgRNA Efficiency and U-Net Analysis

Short guide RNAs were designed with CHOPCHOP and CRISPRscan (https://chopchop.cbu.uib.no; https://www.crisprscan.org) and synthesized by the PCR-based method.^50^ KOD Hot Start DNA Polymerase (Merck, 38018454) was used for amplification, and PCR products were purified with Gel/PCR DNA Fragments Extraction Kit (Faust, 4.661 771). RNA was synthesized with the MEGAscript T7 Transcription Kit (Ambion, AM1333) and cleaned up with the mirVana miRNA Isolation Kit (Ambion, AM1561). Multiple sgRNAs were designed and injected in parallel; genomic DNA was extracted from five embryos (in three technical replicates) using lysate buffer (50mM Tris-HCL pH 8.8, 1mM EDTA and 0.5% Tween-20 in nuclease-free water) together with 20 *µ*g of proteinase K (Fisher Bioreagents, BP1700-100); the gRNA targeted region was amplified using KOD (primer information in Supplemental Table 8); primer residues were purified with a ExoSAP-IT PCR Product Cleanup Reagent (Life Technologies, 78200.200); and products were Sanger-sequenced. Efficiency was calculated using ICE CRISPR analysis (https://ice.synthego.com/) (Supplemental Figure 6C) comparing injected and WT embryos from the same clutch.

Tadpoles were fixed in MEMFA, bleached with 10% H_2_O_2_ (VWR, 1.07209.0500) in methanol, and stained with lectin (described under stainings) to visualize the pronephros. Images were acquired with a stereomicroscope and processed using a Fiji^51^ U-Net deep learning pipeline^52,53^ for automatic and unbiased quantification of kidney sizes. In brief, a pretrained model for quantifying kidney sizes in lectin-stained *X. tropicalis* embryos was fine-tuned using a transfer learning approach to perform accurately on this imaging dataset (IOU: 0.83). The kidney area changes were calculated comparing U-Net measured areas of the uninjected side with the injected side within every tadpole, and pairwise ANOVA was conducted to evaluate the statistical significance of the manipulation.

For *X. tropicalis* MesoSPIM imaging, embryos were double stained for LE-lectin and Atp1a1 (DSHB a5) embedded in 2% low-melting agarose and dehydrated as follows: 75% MeOH/25% 1× PBS (15 minutes), 50% MeOH/50% 1× PBS (15 minutes), 25% MeOH/75% 1× PBS (15 minutes), and three times 100% MeOH (45 minutes each). Clearing was performed in BABB (benzyl alcohol:benzyl benzoate 1:2) overnight. The samples were imaged using selective plane illumination microscopy (MesoSPIM).^54^ For all MesoSPIM recordings, fluorophores were excited with the appropriate laser lines and the quadband emission filter (BP444/27; BP523/22; BP594/20; BP704/46) was used.

#### Induction of Renal Tissue in Ectodermal Explants (Animal Caps)

*X. laevis* embryos were obtained by *in vitro* fertilization. The jelly coat was removed with 3% L-Cystein (BioChemica, A3694,0100) diluted in 0.3X MMR. For injections, fertilized eggs were transferred to 3% Ficoll (Milian SA, 17-0300-10) diluted in 0.1X MMR. All four blastomeres were microinjected at the four-cell stage with 0.1–0.2 ng of mRNA encoding the respective protein. The vitelline membrane was removed, and animal caps were isolated at stage 9 (Nieuwkoop and Faber^49^). The explants were cut with an “eyebrow knife,” washed several times, and kept in a Steinberg solution until uninjected control embryos reached stage 40 (2 days and 18 hours at 23°C).

### Stainings

#### Animal Cap Stainings

Animal caps explants and tadpoles from the same clutch were fixed with MEMFA fixative (1/10 10xMEM salts, 1/10 37% formaldehyde and 8/10 H_2_O) and blocked with 0.1% Triton X (ROTH, 3051), 0.2% BSA (Biomol, 01400.100), and 20% goat serum (Sigma-Aldrich, G9023) in PBS. Kidney-specific antibodies 3G8 and 4A6 from the European Xenopus Resource Center (EXRC) were used at dilutions of 1:10 and 1:4, respectively. For the secondary antibody, Cy3 AffiniPure Donkey Anti-Mouse IgG (Jackson ImmunoResearch, AB_2340813) was used at a dilution of 1:500.

Animal caps were embedded in Technovit 7100 and 3-*µ*m thin sections made with a microtome. Samples were treated as previously described.^48^ Sections were stained with eosin and DAPI following standard protocols.

#### Whole-Mount *In Situ* Hybridization

For *in situ* hybridization, antisense RNA probes were generated by *in vitro* transcription with T7 (Sigma-Aldrich, 10881767001) and labeled with digoxigenin (Sigma-Aldrich, 11093657910) as described.^55^ The plasmids used to generate the *sglt1* and *nkcc2* probes were a gift from Oliver Wessely^56^ and Peter Vize.^46^ For detection, an alkaline phosphatase–conjugated antibody against digoxigenin was used (Roche, 11093274910). To characterize also the phenotype next to *in situ* staining in hnf1b crispants, tadpoles were stained with *Lycopersicon esculentum* (LE-)lectin (Vector Laboratories, DL-1174-M001) 1:100 in a blocking solution (0.15 M NaCl, 0.01 M Tris pH 7.5, 10% FBS, and 5% DMSO diluted in water) overnight. Images were acquired using Zeiss Discovery.V8 and Zen2011 Blue Edition software.

#### Immunostainings

Biological replicates of HNF1B WT and HNF1B R295C reprogrammed cells were washed with ice-cold PBS and fixed in 4% PFA (Santa Cruz Biotechnology, sc-281692) for 20 minutes at room temperature. Cells were permeabilized for 15 minutes in PBST (0.5% Triton X-100 in PBS) and blocked 2 hours with 5% normal goat serum (abcam, ab7481) and 1% BSA (MP Biomedicals, 0216006980) in PBST (blocking solution). Primary and secondary antibodies were incubated overnight and 2 hours, respectively. DNA of fixed cells was stained with 1:4000 dilution of Hoechst 33258 (Cayman, Cay16756-50) in PBS. Primary and secondary antibodies were diluted in the blocking solution. Antibodies and dilutions used for immunofluorescent staining are provided in Supplemental Table 9. Samples were mounted with an Ibidi mounting medium (Ibidi, 50001) and analyzed using a Leica DMI 6000 fluorescence microscope or an Olympus spinning disk for 3D cultures.

#### Immunostainings in the Mouse Kidney

Briefly, kidneys of isoflurane-anesthetized mice were fixed by perfusion of the aorta abdominalis with 3% paraformaldehyde (PFA), followed by a rinsing step with 0.1 M phosphate buffer (pH 7.4, 250 mOsm) and frozen in liquid propane until further usage. Cryosections of 5-*µ*m thickness were cut at −19°C and temporarily stored in 1× PBS for immunohistochemistry. After blocking for 3 hours at room temperature with 10% goat serum (Abcam, ab7482) and 0.2% Tween20 (PanReac AppliChem, A4974) in 1× PBS, slices were incubated overnight at +4°C with a primary antibody dilution or 1× PBS (controls). Subsequently, samples were incubated 3 hours at room temperature with a secondary antibody dilution containing 1:1000 DAPI (Thermo Fisher Scientific, D1306), for a final concentration of 1 *µ*g/ml. Slides were mounted with the Ibidi mounting medium (Ibidi, 50001) and analyzed under the Leica DMI 6000 fluorescence microscope. Slices were washed three times in 1× PBS in between critical steps. Primary and secondary antibodies were diluted in 1x PBS containing 10% blocking solution. All dilutions are reported in Supplemental Table 9.

### Quantitative PCR

RNA was extracted in triplicates from p22 HNF1B WT and R295C iRECs. In particular, each replicate was one clone of HNF1B WT and the corresponding HNF1B R295C. Total RNA was extracted using the QIAzol Lysis Reagent (Qiagen, no. 79306) and isolated with the RNeasy Plus Universal Mini Kit (Qiagen, no. 73404), according to the manufacturer's instructions. One *µ*g of total RNA was reversely transcribed to cDNA with the QuantiTect Reverse Transcription Kit (Qiagen, no. 205311). Quantitative PCR was performed on a Roche LightCycler 480 instrument using a Light Cycler 480 SYBR Green I Master (Roche, no. 04707516001). Per reaction, 10 ng of cDNA template were applied with primers listed in Supplemental Table 10. Threshold cycle (CT) values were normalized to CT values of Tbp (TATA box-binding protein) as a housekeeping gene and analyzed applying the comparative CT method.^57^

### RNA Sequencing

For *Xenopus* explants, 20 biological replicates were pooled, and RNA was extracted with the RNeasy Mini Kit (Qiagen, 74104) and purified with ethanol precipitation. For mouse cells, RNA was extracted from one confluent dish with the RNeasy Plus Universal Mini Kit (Qiagen, 73404).

### Data Analysis

RNA from three replicates of reprogrammed iRECs and induced explants from both WT and HNF1B R295C conditions were sequenced. As a control condition, RNA from original MEFs and animal cap tissue was sequenced. All the samples were analyzed on a Galaxy platform^58^ using FastQC v0.11.5 for quality control, Trim Galore! version 0.4.3 for trimming, STAR v2.5.2b-2^59^ for alignment (with genome assemblies GRCm38 and *X. laevis* v. 9.2), featureCounts v1.6.0^60^ for counting the reads, and DESeq2 v1.18.1^61^ for differential expression analysis. From the results of PCA analysis using PCAtools,^62^ the WT2 sample was eliminated from further analysis because it clustered most closely with the uninjected samples. All the DESeq2 results for three different comparisons for mouse data are available in Supplemental Table 1 and for *Xenopus* data in Supplemental Table 2. *P* values were adjusted with the Benjamini-Hochberg multiple testing, and for significantly differentially expressed genes, a cutoff of *P*-adj<0.05 was used.

### Clustering

Genes were first clustered into 12 groups with the soft clustering tool Mfuzz.^63^ Outliers were discarded with a *posteriori* filtering method using *α*-threshold 0.9 for the gene's membership value in the group. The groups were then assigned to their corresponding clusters on the basis of the expression pattern changes.

### Downstream Analysis

To explore these gene clusters and their functional profile, the GO overrepresentation test was performed using Bioconductor^64,65^ package clusterProfiler^66^ v3.10. Using comparison analysis, profiles were computed for every gene cluster representing biological processes, cellular components, and molecular functions enriched most within the cluster.

For visualization, R packages ggplot2,^67^ Heatmap2,^68^ ComplexHeatmap,^69^ VennDiagram,^70^ and EnhancedVolcano^71^ were used. Text and data frame manipulations were performed using Hmisc,^72^ Tidyverse,^73^ and reshape2.^74^ To analyze overlap between species, biomartr^75^ and Xenbase^76^ orthology information was used. To evaluate the molecular functions, expression patterns, and pathology of the target genes gnomAD,^20^ GTEx^77^ and Hnf1b target genes and the distances of the transcription starting site were extracted from the ChIP-Atlas (*M. musculus* version 9 dataset).^78^ Collecting duct, distal tubular and proximal tubular cell enhanced genes were extracted from the Human Protein Atlas' dataset The Single Cell Type Atlas.^79^ The HNF1B crystal structure was retrieved from the protein database (PDB accession: 2H8R).^29^ R295C mutation was modeled *in silico* using the PyMol mutagenesis tool, selecting the most favorable rotamer.

## Results

### Transcription Factors Can Induce Renal Tissue in Ectodermal Explants

**Figure 1:**
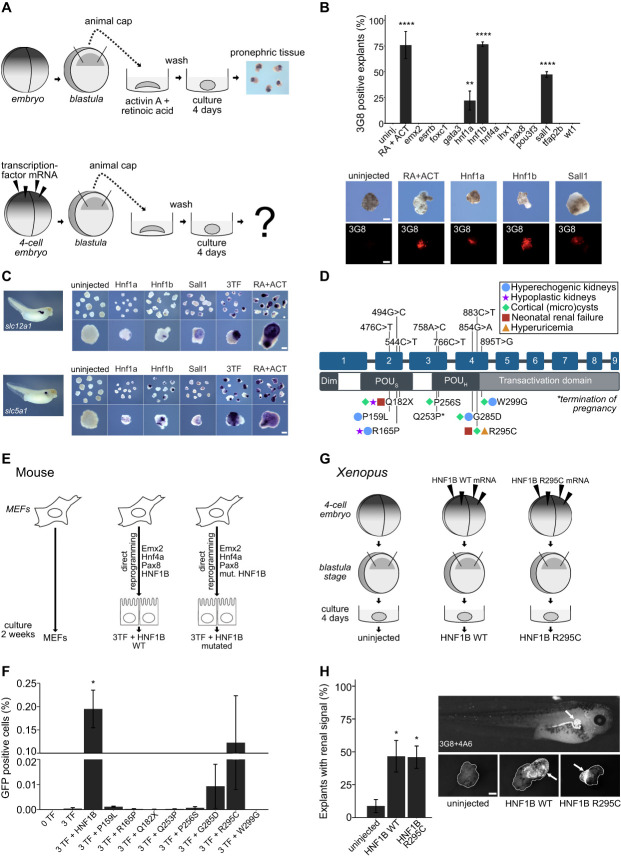
**Renal tissue induction in *Xenopus* ectodermal explants and mouse embryonic fibroblasts using transcription factors.** (A) Schematics of the induction of pronephric tissue from *Xenopus* animal caps. Induction of pronephric tissue from animal caps treated with retinoic acid (RA) and activin A (ACT) has been previously shown by Moriya *et al.* (B) Results of induction efficiency using 13 renal organogenesis transcription factors and RA+ACT treatment compared with untreated (uninj.). Explants with a positive signal for the proximal tubule marker (3G8) are illustrated in the images underneath. (C) *In situ* stainings of distal (*slc12a1/*Nkcc2) and proximal (*slc5a1/*Sglt1) renal markers in explants induced by expression of Hnf1a, Hnf1b, Sall1, or all three factors combined compared with RA+ACT and uninjected. (D) Patient-specific mutations and the postnatal renal phenotypes illustrated on the HNF1B gene/protein schematic. (E) Schematics of the reprogramming workflow. Mouse embryonic fibroblasts were directly reprogrammed to induced renal tubular epithelial cells (iRECs) using four transcription factors (TFs)—Emx2, Hnf4a, Pax8, and HNF1B. To investigate HNF1B and its clinically relevant mutations, reprogramming with mutated HNF1B was conducted. (F) Reprogramming efficiency with patient-specific mutations. (G) Induction of pronephric tissue from animal caps with HNF1B WT and HNF1B R295C mRNA injections. (H) Proximal (3G8), distal, and connecting tubule marker (4A6) stainings in explants and a *Xenopus* tadpole of the same age. Positive stainings are highlighted with an arrow. Mean percentage of explants showing staining for renal markers in uninjected (*n*=99), injected with *HNF1B* WT (*n*=61), and explant injected with *HNF1B* R295C (*n*=91). Error bars, SEM; asterisks indicate significant differences to the positive control (uninj (B), 0TF (F) and uninjected (H)) as assessed by pairwise *t*-tests with corrections for multiple testing (Bonferroni), biological replicates *n* = 3–5 (B), *n*=3 (F), *n*=7 (H), ****P<0.0001, ***P<0.001, **P<0.01, *P<0.05. All scale bars are 200 *µ*m.

We previously analyzed the expression pattern of transcription factors in *Xenopus* embryos that have high and specific expression in mammalian adult kidneys.^39^ We focused on 13 factors with a strong expression during renal organogenesis (Emx2, Esrrb, Foxc1, Gata3, Hnf1a, Hnf1b, Hnf4a, Lhx1, Pax8, Pou3f3, Sall1, Tfap2b, Wt1) and expressed them by injection of mRNA into four-cell stage *Xenopus laevis* embryos (Figure 1A). Ectodermal explants (animal caps) were obtained pregastrulation and cultured until control embryos had developed a functional pronephros (Nieuwkoop and Faber [NF]^49^ stages 37–40). Renal tissue was detected by immunostaining in control explants treated with activin A and retinoic acid. Explants expressing *hnf1b* and to a lesser extent *hnf1a* or *sall1* also developed ectopic renal tissue, but not any of the explants expressing one of the other ten factors, nor uninjected control explants (Figure 1B). To confirm the presence and segment identity of the ectopic renal tissue, explants were subjected to *in situ* hybridization, which detected transcripts of *slc5a1* (Figure 1C and Supplemental Figure 1A; encoding the proximal tubule marker Sglt1) and *slc12a1* (Figure 1C; encoding the intermediate loop and early distal tubule marker Nkcc2), confirming the presence of differentiated tubular tissue in the explants injected with *hnf1a*, *hnf1b*, and *sall1* mRNAs individually. Combined expression of these three factors increased the *in situ* signal for both markers, suggesting that while each factor is sufficient to induce renal embryonic organoids in *Xenopus*, their combined expression can lead to synergistic effects.

### The Patient Mutation R295C Maintains the Ability to Induce Renal Tissue

Next, we explored how mutations in *HNF1B* responsible for causing renal malformations in patients would affect protein function using the two *de novo* tubule induction models. Therefore, we tested whether patient-specific mutations would impair the direct reprogramming ability of HNF1B. Eight patient-specific point mutations in *HNF1B* causing developmental renal abnormalities^6,80,81^ were introduced into lentiviral expression constructs (Figure 1D). Lentiviral expression of wild-type (WT) HNF1B and the eight variants containing the selected mutations in combination with the three additional factors Emx2, Hnf4a, and Pax8 (3TF) reprogrammed mouse embryonic fibroblasts (MEFs) into iRECs. MEFs were derived from double transgenic animals (Ksp:Cre;mTmG) expressing Cre recombinase under the control of the kidney-specific cadherin-16 promoter and a dual fluorescent reporter (tdTomato/EGFP), in which Cre-mediated recombination causes a switch from tdTomato to GFP expression on successful reprogramming (Figure 1E). The frequency of reporter activation (% GFP-positive cells) was used to quantify reprogramming efficiency after a maximum of 28 days in culture. WT HNF1B and HNF1B harboring a missense mutation leading to an arginine-to-cysteine change in position 295 (c.883C>T) plus 3TF were able to generate reprogrammed cells (Figure 1F). The mutations P159L, P256S, and G285D also led to a detectable increase in GFP-positive cells, albeit at very low levels. No significant reporter activation was detected for the mutations R165P, Q182X, Q253P, and W299G.

Likewise, expression of HNF1B R295C was also able to induce renal-like tissue in *Xenopus* ectodermal explant cultures (Figure 1, G and H), confirming that the mutated protein retained the protein function necessary for induction of the nephrogenic program. Expression analysis by Western blot confirmed similar protein expression of the WT and R295C mutant HNF1B protein (Supplemental Figure 1B).

**Figure 2:**
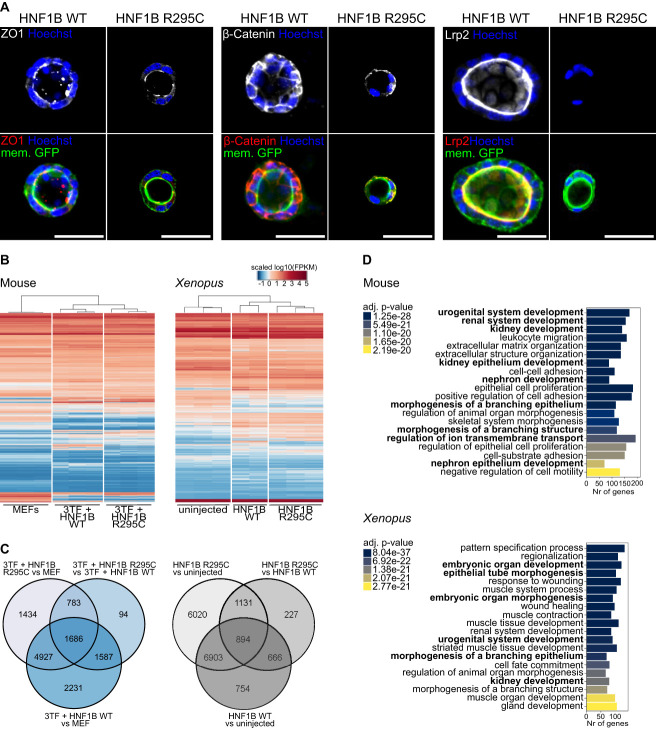
**Profiles of cells reprogrammed with HNF1B WT and HNF1B R295C.** (A) Immunofluorescence stainings of HNF1B WT and R295C iRECs grown in 3D Matrigel for epithelial markers. Merged images consist of iRECs membrane GFP (green), target protein (red), and Hoechst (blue) signals. All scale bars are 50 *µ*m. (B) Heatmap (left) of all the genes with significantly changed expression in at least one of the comparisons (MEFs versus 3TF+HNF1B WT, MEFs versus 3TF+HNF1B R295C, 3TF+HNF1B WT versus 3TF+HNF1B R295C) in mouse fibroblast reprogramming (*N*=12,742), and heatmap (right) of all the genes significant in at least one of the comparisons (uninjected versus HNF1B WT, uninjected versus HNF1B R295C and HNF1B WT versus HNF1B R295C) in explant inductions (*N*=16,595). (C) Venn diagrams of significantly differentially expressed (DE) up- and downregulated genes between comparisons in mouse reprogramming (blue) and explant induction (gray). (D) GO over-representation test on DE genes (absolute log_2_ fold change >1.5) from reprogramming experiments comparing 3TF+HNF1B WT iRECs with MEFs (top) and HNF1B WT pronephric tissue with uninjected animal caps (bottom). GO terms related to renal organogenesis are marked in bold. The same analysis for the R295C mutation condition is showed in Supplemental Figure 2A.

To characterize our *in vitro* model in more depth, WT HNF1B and HNF1B R295C reprogrammed cells were cultured in 3D Matrigel. Up to 10 days later, we identified spheroids in both conditions and confirmed correct apicobasal polarization by detecting signals for the epithelial markers ZO1, β-Catenin, and Lrp2 (Figure 2A).

Distinct point mutations at R295 (R295H, R295P) have been previously mapped onto the HNF1B crystal structure scaffold and were experimentally shown to alter but not abolish DNA-binding affinity.^29^ Mapping of the R295C mutation similarly revealed the loss of a salt bridge to the adenines at positions 11 and 12 of the canonical DNA-binding motive of HNF1B, but did not impair DNA binding as verified by a DNA mobility shift assay (Supplemental Figure 1C). Although WT and R295C mutant HNF1B displayed a similar expression level, the protein stability over time was slightly reduced in the R295C mutant as validated by a cycloheximide chase assay (Supplemental Figure 1D–F). This suggested that owing to a slightly reduced protein stability, the transcriptional activation may be altered.

In conclusion, HNF1B R295C retained the DNA-binding activity sufficient for direct reprogramming of iRECs and could also induce renal-like tissue in *Xenopus* explant cultures. This provided a unique opportunity to test whether specific target genes are affected by a slightly altered protein stability of the R295C substitution and identify these.

### Cells Reprogrammed by HNF1B R295C Have Distinct Changes in Their Transcriptional Profile

To investigate the changes in transcriptional activity due to the R295C mutation in *HNF1B*, three replicates of reprogramming were conducted using both WT and R295C HNF1B. GFP-positive reprogrammed cells (iRECs) were sorted and expanded, and RNA was extracted from these groups together with control MEFs for RNA sequencing (Figure 2B).

Across all three samples, the expression of 12,742 genes was significantly different in at least one of the comparisons (MEFs versus 3TF+HNF1B WT, MEFs versus 3TF+HNF1B R295C, 3TF+HNF1B WT versus 3TF+HNF1B R295C) (Figure 2B). Pairwise comparison revealed that most transcriptional changes (10,431 differentially expressed genes) occurred between MEFs and iRECs while only 4150 genes were differentially expressed between iRECs reprogrammed using WT or R295C HNF1B (Figure 2C, Supplemental Table 1 and Supplemental Table 2). Gene ontology (GO) analysis confirmed that both WT and R295C HNF1B reprogrammed iRECs were enriched for genes associated with renal function, as compared with MEFs, confirming that successful reprogramming to renal-like cells had occurred (Figure 2D).

In analogy to the experimental setup detailed above, we also isolated RNA from ectodermal explants overexpressing WT and R295C HNF1B and a control group of uninjected explants (Figure 1A). All samples segregated well into the expected clusters after RNA sequencing, with the exception of one sample derived from WT HNF1B-injected explants (WT2), which was indistinguishable from uninjected controls, and therefore excluded from the analysis (Supplemental Figure 2B). Overall, the absolute changes in RNA levels were not as strong as seen in the reprogrammed cells, likely because expression of transcription factors only converts part of the explants to pronephric tissue. Nevertheless, we observed clear differences between the groups, most strikingly between the uninjected group and those that received either WT or R295C HNF1B injections, as seen in the iRECs model. GO analysis of differentially expressed genes between HNF1B and uninjected explants revealed that many of the altered changes are consistent with renal organ development (Figure 2D), confirming the potential of HNF1B to drive noncommitted cells toward a renal fate.

A subset of genes had a significantly changed expression in iRECs reprogrammed with WT HNF1B compared with R295C and were also differentially regulated for MEFs. We also detected distinct clusters of genes that were differentially regulated when WT and R295C HNF1B-induced *Xenopus* explants were compared (Figure 2, C and D). Thus, we observed in two independent experiments and species that a distinct group of genes was affected by the patient mutation while the overall transcriptional activity was unchanged for most of HNF1B downstream targets. This confirmed that the R295C mutation in HNF1B affects the transcriptional activation or repression of specific target genes, as opposed to having global effects on its transcriptional activity *per se*.

**Figure 3:**
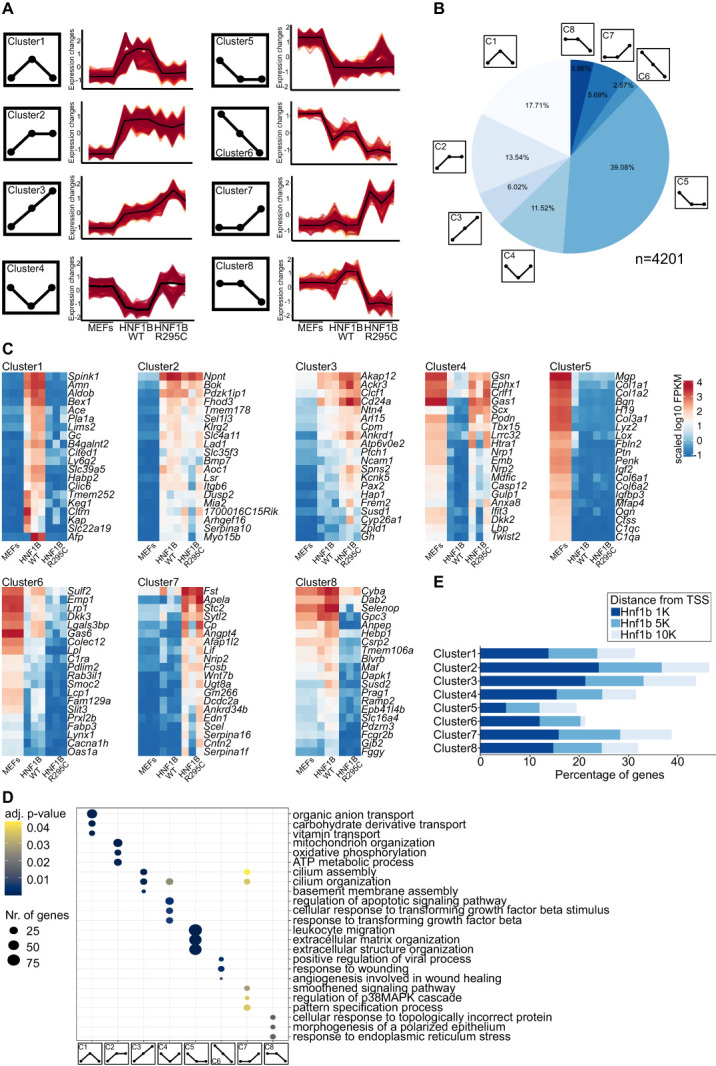
**Transcriptional alterations due to HNF1B patient-specific mutation R295C**. (A) Clustering DE genes according to expression patterns. Schematics represent the eight expected expression patterns while line graphs show the expression changes in the mouse transcriptome data. (B) Pie chart of the distribution of the genes in all clusters (4201 genes in total). (C) Heatmaps of top 20 most variable (variance to the mean of the cluster) genes within each cluster. (D) Comparing clusters on the GO biological process level and visualizing top three processes per cluster (full plot in Supplemental Figure 3A). (E) Percentages of genes per cluster with 1, 5, or 10-kilo base pair (K) distance from the calculated transcription starting site (data from ChIP-Seq atlas^78^).

To evaluate these differentially expressed genes in more detail, we used a soft clustering technique to identify groups of genes with a shared expression pattern across samples. Because of the higher homogeneity and sensitivity to detect transcriptional changes in mouse reprogrammed cells, we focused our analysis on this dataset (Figure 3). After filtering for a membership value *α*-threshold over 0.9, we assigned a total of 4201 genes to 12 groups by soft clustering (Supplemental Figure 2C).

Each of the groups was then assigned to one of eight clusters reflecting all possible pattern changes between the analyzed samples (Figure 3A). With 1642 genes, cluster 5 was the biggest cluster representing genes equally downregulated in both WT and R295C HNF1B reprogrammed iRECs compared with MEFs (Figure 3B). Direct and indirect targets transcriptionally activated by HNF1B and affected by the mutation are most likely to fall into cluster 1 (*N*=744). The R295C mutation does not seem to impair DNA binding (Supplemental Figure 1C). Nevertheless, targets from cluster 1 are not activated by the HNF1B R295C protein. By contrast, cluster 4 (*N*=484) contains genes repressed by HNF1B, which are no longer repressed in iRECs reprogrammed with HNF1B R295C (all clusters and genes in Supplemental Table 3). The 20 most variable genes for each cluster are shown as a heatmap (Figure 3C). Thus, we detected changes to both activating and repressing functions conferred by the R295C mutation.

To explore the functional profiles of these clusters, GO overrepresentation tests were performed. Cluster 1 genes are associated with different cell transport processes and the brush border (Figure 3D and Supplemental Table 4). These processes are highly relevant to the function of proximal tubular cells.^82^ This is consistent with the known role of HNF1B in controlling the specification of the proximal intermediate nephron segment^83^ and its impact on regulating numerous transport processes.^84^ Surprisingly, cluster 3 (similarly to clusters 4 and 7) representing genes upregulated in reprogrammed cells and having even higher expression levels in HNF1B R295C reprogrammed cells, consisted of genes associated with cilia, for example *Ahi1*, *Cep290*, *Ift80*, *Foxj1* (Figure 3D). HNF1B target genes play important roles in primary cilium formation and maintenance,^85,86^ and ciliary dysfunction can cause renal cysts.^87,88^ Our data suggest that HNF1B may have a repressive function on these target genes that is lost by the R295C mutation. The full lists of GO results from all three levels (biological processes, molecular functions, and cellular components) are provided in Supplemental Figure 3A. To validate the clusters against experimentally established HNF1B target genes, we compared each cluster with ChIP-Seq datasets (ChIP-Seq atlas,^78^ Figure 3E). On average, 33% of clustered genes had a potential Hnf1b-binding site (peak-call data) within 10 kbp of the transcriptional start site. In 15% of genes, it was closer than 1 kbp to the transcriptional start site, which may represent direct HNF1b targets. However, because the available ChIP-Seq analysis was conducted on distal tubular cells, genes highly expressed in proximal segments may be underrepresented. Therefore, many of the differentially regulated genes we observed, for which no direct binding has been documented, may nevertheless represent veritable targets predominantly expressed in proximal tubule cells. The high proportion of GO terms related to proximal tubular function (Figure 3D) supports this conclusion.

### HNF1B R295C Changes the Expression of Kidney Segment-Specific Genes

We next analyzed what effect HNF1B WT and R295C has on the expression of marker genes for nephron segments using single-cell RNA sequencing data from the Human Protein Atlas (Supplemental Figure 3B). Most prominently, genes specific for the proximal tubule were more downregulated in cells reprogrammed with HNF1B R295C as compared with iRECs generated with the WT protein. Although some genes associated with the distal tubular segment identity also showed significant changes, this was less pronounced than seen for the proximal tubules. Of the 315 genes with enhanced expression in proximal tubular cells that were differentially expressed between iRECs reprogrammed with HNF1B WT and R295C, more than 80% were downregulated. By contrast, 60% and 48% of 220 distal tubular and 180 collecting duct genes, respectively, were downregulated (Supplemental Figure 3C).

To evaluate whether these changes were stable, we measured the expression of a set of tubular differentiation markers in iRECs of later passages (passage 22) by qPCR. *Cdh6* and *Cdh16* maintained their high expression levels. Consistent with the RNA sequencing results, *Lrp2* and *Slc17a1* were only significantly induced by WT, but not R295C HNF1B (Supplemental Figure 4A). Thus, the transcriptional changes observed seem stable over longer periods in culture.

### Transcriptional Changes in Known Kidney Disease Genes and Transcription Factors

**Figure 4:**
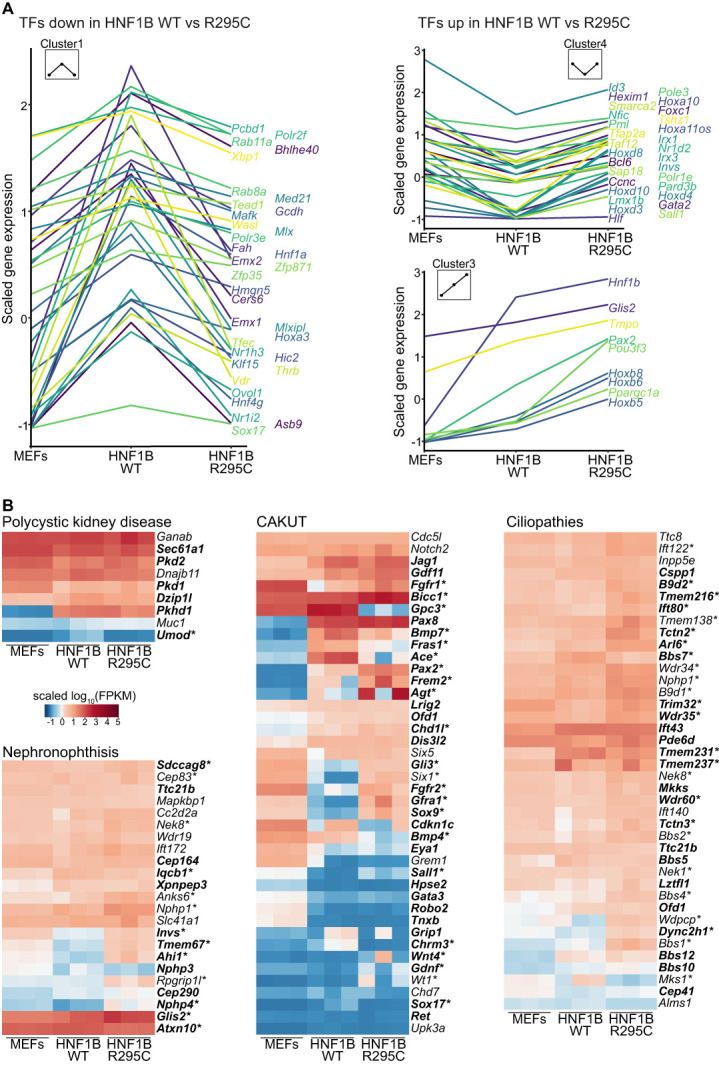
**Changes in transcription factors and genes related to kidney disease.** (A) Expression patterns of transcription factors involved in kidney disease. Line graphs represent mean expression changes in three replicates along the three conditions; the plots were divided to capture the significant changes between HNF1B WT and R295C conditions. (B) Expression profile of disease-associated genes in fibroblasts and reprogrammed tissue. A significant difference in the expression levels comparing 3TF+HNF1B WT versus MEFs are represented in bold; asterisk (*) indicates a significant difference between 3TF+HNF1B R295C versus 3TF+HNF1B WT.

Induction and control of kidney development is orchestrated by a network of transcriptional regulators. Analysis of this group of genes revealed a large number of downregulated genes in the R295C condition, including *Hnf1a* and *Pcdb1*, two cofactors of HNF1B (Figure 4A). However, this list also includes other transcription factors that may relay transcriptional control of nephrogenic targets downstream of HNF1B.

HNF1B and many of its targets are known to cause inherited kidney disease, including CAKUT, and cystic dysplastic kidneys. In addition, genes associated with recessive polycystic kidney disease and autosomal cystic kidney disease have been identified as transcriptional targets of Hnf1b. Given the link between HNF1B and genetic kidney diseases of various manifestations, we explored the expression of genes associated with inherited kidney disease systematically (Figure 4B). First, we selected genes with a known role in hereditary kidney disease on the basis of public databases.^89–92^ Next, we analyzed their expression in MEFs and iRECs reprogrammed with HNF1B WT or HNF1B R295C. Most of the transcriptional changes were detected in genes related to CAKUT. Interestingly, we also detected upregulation in numerous ciliopathy genes, consistent with cilia-related findings in the cluster analysis.

### Known and Novel HNF1B Targets Identified from Transcriptomes

**Figure 5:**
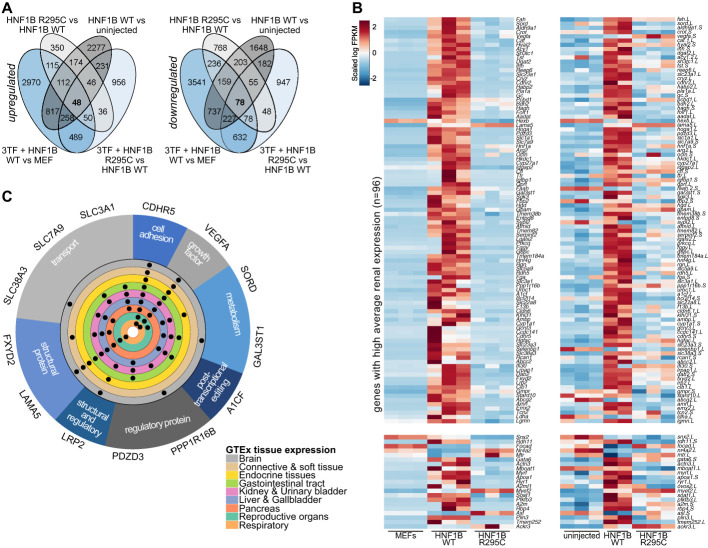
**Species overlap analysis**. (A) Overlap of the DE genes in mouse reprogramming and *Xenopus* renal tissue induction experiments. Blue ellipses indicate reprogramming from fibroblasts (MEF) with 3TF+HNF1B WT or 3TF+HNF1B R295C and the DE comparisons; gray ellipses indicate induction from animal cap (uninjected) with HNF1B WT or HNF1B R295C and the DE comparisons. (B) Heatmap of overlapping DE genes (*N*=117) in both species, separated on the basis of kidney-specific tissue expression in the top part (*N*=96). (C) Validated target genes with their function and tissue expression profile in human tissues (expression data from GTEx^77^).

Expression of HNF1B alone activated a nephrogenic program in the plastic environment of the *Xenopus* explant cultures. Thus, transcriptional profiling of ectopic renal organoids in comparison with the uninjected explants allowed us to isolate HNF1B-dependent activated or repressed programs more directly (Supplemental Figure 4B). In agreement with the reprogramming results in mammalian cells, we detected particularly high induction of genes related to cystic kidney disease, such as *bicc* and *pkd2*. In addition, comparing the datasets obtained from mouse and *Xenopus* experiments allowed us to identify core transcriptional targets of Hnf1b that are conserved throughout vertebrate evolution. We stringently filtered out genes that had the same expression pattern throughout all three conditions in both species (Figure 5A). This resulted in 126 high-confidence Hnf1b target genes, which are also dysregulated by the R295C mutation and thus most likely share a common regulatory mechanism. Of these, 82% were expressed at higher levels in the kidney than in other organs (Figure 5B).^93^ This confirms that the main transcriptional changes detected in our analysis uncover transcriptional targets with a function in the kidney. It is also in agreement with the clinical observation that patients affected by mutations in HNF1B suffer predominantly from renal malformations and to a lesser extent from pancreatic defects or extrarenal pathologies.^6,11,94^

We selected 36 of these candidate target genes on the basis of their high expression in the mammalian kidney or established a role in kidney disease to be functionally evaluated in *Xenopus tropicalis* (Supplemental Table 5). We found 67% of genes to be expressed during embryonic renal development, and a majority showed the strongest expression in the proximal tubular segment (Supplemental Figure 5A). We also detected endogenous *hnf1b* mRNA expression during early pronephros morphogenesis (NF 22) by *in situ* hybridization, in early tubules (NF 33) and in the functional pronephros (NF 38) in *Xenopus* embryos (Supplemental Figure 5B). With the exception of *lama5*, we did not detect a distinct expression of these targets at neurula or early tailbud stages, where hnf1b expression commences and the renal field is specified. Thus, the targets identified in our analysis seem to be involved in renal tubular differentiation and maintenance, rather than in early cell-fate decisions.

**Figure 6:**
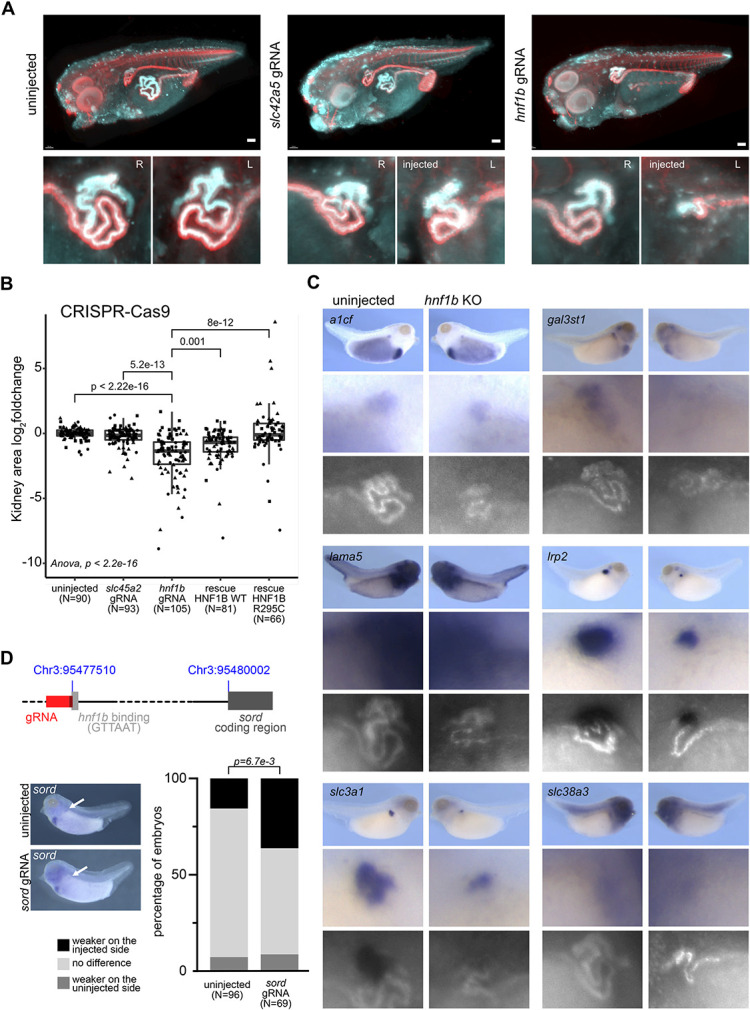
***In vivo* analysis of target genes.** (A) MesoSPIM images of the uninjected (left), control *slc42a5* knockout (KO) (middle), and *hnf1b* KO (right) *Xenopus tropicalis* tadpoles and the kidney area for right (R) uninjected and left (L) manipulated sides. (B) Rescue of the *hnf1b* KO using WT and R295C *HNF1B* mRNA compared with control *slc45a2* KO and uninjected conditions. Log_2_ changes of the kidney area from injected to uninjected sides are plotted for three different experiments (dot shape) and pairwise multiple comparisons (ANOVA) show the significance between uninjected, control injections (*slc45a2* gRNA) and targeted injections. (C) *In situ* hybridization combined with kidney-specific antibody (lectin) stainings of six target genes in WT (left) and CRISPR/Cas9 KO (right) *Xenopus* embryos. (D) *In situ* hybridization to evaluate *sord* mRNA expression in CRISPR/Cas9 experiments with a gRNA (red) targeting the *sord* promoter at the hnf1b binding motive (light gray). Intensity of the staining was scored as weaker or stronger on the injected side or equal on both sides. Significance was calculated with the chi-square test. Scale bar is 100 μm.

To further investigate HNF1B targets affected by the R295C substitution, we focused on 13 genes selected on the basis of renal expression in *Xenopus* tadpoles, expression in human tissue (GTEx^77^), loss-of-function intolerance (gnomAD^20^), and known renal function (*A1cf*, *Cdhr5*, *Gal3st1*, *Fxyd2*, *Lama5*, *Lrp2*, *Pdzd3*, *Ppp1r16b*, *Slc3a1*, *Slc38a3*, *Slc7a9*, *Sord*, *Vegfa*) (Figure 5C). We analyzed whether the endogenous expression of these potential targets would indeed depend on the presence of hnf1b. Therefore, we depleted *Xenopus* embryos unilaterally of *hnf1b* using CRISPR/Cas9 genome editing, which resulted in a shortened, stunted hypoplastic kidney on the injected side visualized by selective plane illumination microscopy (MesoSPIM) (Figure 6A). The phenotype in crispants was rescued by coinjection of mRNA-encoding *HNF1B* (Figure 6B). It was also rescued by coinjecting mRNA-encoding HNF1B R295C consistent with our previous findings that this variant retains most of the WT function, in particular by activating the nephrogenic specification network and alters only the expression of a select number of genes predominantly active in the differentiated tubule. Thus, CRISPR depletion of hnf1b was specific and subsequently used to determine the endogenous role for hnf1b to activate the transcription of potential target genes.

Next, we tested the expression of the 13 target genes in *hnf1b* crispant tadpoles by *in situ* hybridization together with the kidney-specific LE-lectin staining (Figure 6C, full analysis in Supplemental Figure 5C). We detected distinct downregulation of all potential targets in the absence of *hnf1b*, including *Lama5*, the only gene marginally upregulated in HNF1B WT versus R295C reprogrammed cells. To further validate the hnf1b transcriptional regulation of potential target genes, we searched for suitable gRNA target sites in the predicted hnf1b-binding motifs in their promoter regions. Targeting the hnf1b-binding motive of the *sord* promoter region (1000 bp upstream from the coding region) with CRISPR/Cas9 decreased its mRNA expression in the pronephros significantly (Figure 6D). These results suggest that *sord* is directly transcriptionally regulated by hnf1b.

### Dysregulation of HNF1B Targets Affects Protein Abundance

**Figure 7:**
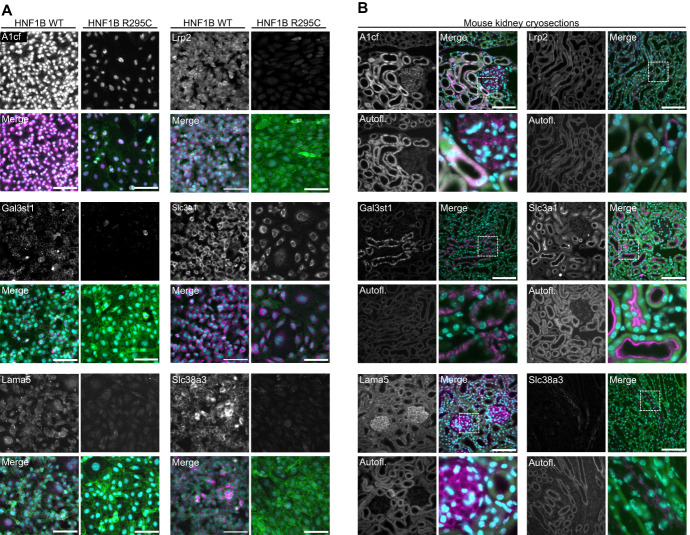
***In vitro* analysis of target genes.** (A) Immunofluorescence staining of HNF1B WT (left) and R295C (right) iRECs for six proteins encoded by Hnf1b-regulated genes. Merged images contain eGFP signals from the iRECs (green), antibody staining, (magenta) and nuclear stain with Hoechst (cyan). Images for all three replicates together with secondary antibodies are presented in Supplemental Figure 6A. (B) Immunofluorescence of six proteins encoded by Hnf1b-regulated genes in mouse kidney cryosections. Merged images contain autofluorescence (autofl.) of the kidney tubules (green), antibody staining (magenta), and nuclear stain with DAPI (cyan). All scale bars are 100 *μ*m.

To cross validate our findings in directly reprogrammed mammalian cells, we performed immunostaining for six proteins encoded by predicted target genes (Figure 7A). Because the renal expression patterns of some of these proteins have not been described, we performed immunostaining on sections of adult mouse kidneys (Figure 7B). All the proteins were located in the cortex of the kidney, except Slc38a3 that was found exclusively in the medulla. A1cf and Lrp2 were found in the glomeruli and proximal tubules. Slc3a1 was exclusively found in proximal tubules located in the brush border. Lrp2 was located in the apical and basolateral membrane of the proximal tubules. Gal3st1 was located in the membrane of the intermediate and distal tubules. Slc38a3 was found in the membrane of collecting tubules. Lama5 was located in the membrane and interstitial space of all parts of the mouse renal tubules.

Immunostaining these proteins in iRECs detected lower expression levels in cells reprogrammed with HNF1B R295C as compared with those reprogrammed with the WT factor while their subcellular localization remained largely unchanged (Figure 7A). These data further confirm that the transcriptional targets downstream of HNF1B we identified are dysregulated by the pathogenic mutation and lead to reduced expression of their protein products.

## Discussion

Combining directly reprogrammed mammalian cells and induction of renal organoids in *Xenopus* tissue allowed us to gain unique insights into the transcriptional effects of a missense mutation in *HNF1B* and identify evolutionary conserved transcriptional targets. Both experimental models robustly activated a nephrogenic program. Thus, both directly reprogrammed cells and embryonic *Xenopus* organoids are models of *de novo* tubule formation, which are even more powerful to uncover core nephrogenic programs when used in combination. Our comparative transcriptomic analysis uncovered many genes known to be regulated by HNF1B^95,96^ but also identified additional genes influenced by the transcriptional activity of HNF1B. In contrast to assays that rely on direct DNA binding of HNF1B to recognition motives, such as Chip-Seq, our approach detects the functional results of altered transcriptional activity. We cannot distinguish direct targets from genes indirectly affected by intermediate transcription factors that are themselves controlled by HNF1B. However, the observed changes to the transcriptome of two vertebrate species uncover genes that are evolutionarily conserved and thus constitute core targets, likely controlled by HNF1B also in humans. The use of the *Xenopus* model allowed us to validate a number of newly identified target genes *in vivo* by loss-of-function CRISPR experiments. Although Hnf1b has previously been shown to induce early nephrogenic transcription factors in *Xenopus* ectodermal explants, we detected ectopic renal tissue expressing additional markers of tubular differentiation.^97^

While a partial loss of function has been attributed to the pathogenicity of HNF1B mutations, the specific transcriptional changes of individual patient mutations in the coding region of *HNF1B* have not been elucidated. The substitution of WT HNF1B with a patient-specific mutant (R295C) identified distinct dysregulated gene clusters in our analysis. This suggests that the alterations in the transcriptional activity of this particular mutation are gene- and possibly context-specific. The amino acid substitution of arginine to cysteine interferes with a salt bridge in proximity to two adenines of the recognition motive.^29^ Substitutions in this site had earlier been postulated to lead to target-gene specific changes in transcriptional activity, consistent with our current observations. Slightly reduced protein stability may contribute to the observed changes. However, genome-wide affinity mapping should resolve how patient mutations affect individual gene regulation more precisely. Our analysis can now pinpoint the target genes most likely affected by the R295C substitution.

HNF1B has multiple roles during various steps of renal development, including controlling early nephrogenesis, nephron segmentation, transport activity, and the metabolic profile of differentiated tubule cells.^98^ Among the dysregulated targets followed up in the embryonic renal development of *Xenopus* tadpoles, we were not able to find genes with an expression pattern that started in early development or shortly after Hnf1b was detectable. Most of the genes identified were active after tubulogenesis and likely function in the differentiated kidney, consistent with recent findings of tubular differentiation defects in a novel mouse model for *Hnf1b* haploinsufficiency.^33^ Thus, the R295C mutation seems to preferentially affect the tubular maintenance or mature tubular functions. However, genes listed here constitute core evolutionarily conserved targets. Intriguingly, many have not been ascribed a role in renal development or physiology and should be subject to further functional evaluation. In addition, it is possible that the R295C mutation alters the relative composition of tubular segment identities within reprogrammed or induced renal tissues.

Interestingly, a number of kidney disease-associated genes were found to be differentially regulated, including cilia-associated genes. A number of known genes that cause ciliopathies when mutated (*e.g., Glis2, Umod, Nphp1*, *Invs*, *Nek8*, *Nphp4*) were found to be dysregulated in our induced cell models while others previously identified as direct Hnf1b targets^99,100^ (*e.g., Pkhd1*, *Pkd2*) did not have a significantly changed transcriptional level.^99,101–105^ Curiously, many of these were derepressed by HNF1B R295C. Previous studies using conditional mouse models did not find structural ciliary defects unrelated to a loss of apicobasal polarity in tubular epithelial cells.^85,99,100,106^ These data suggest that the degree of ciliopathy pathway dysregulation in HNF1B disease may extend to additional cystogenic genes, but will require further *in vivo* confirmation experiments.

Among the conserved targets of HNF1B is *Gal3st1*, a galactosylceramide sulfotransferase essential for the biogenesis of sulfatide glycolipids at the outer sheet of the plasma membrane. *Gal3st1* is also a hypoxia-responsive gene that can be upregulated by both HIF1α and HIF2α in the state of hypoxia.^107^ Sulfatides in the kidney are important for adaptation to metabolic acidosis, which can occur during hypoxia.^108^ However, HNF1B itself is also activated by hypoxia, independently from HIF-1ɑ.^109^ Hypoxia signaling is strongly activated in cystic kidney disease and promotes cyst growth through chloride secretion.^110^ However, stabilized HIF-1ɑ occurred in a fumarate hydratase (FH1) knockout mouse, which also developed renal cysts.^111^ Likewise, we detected FH1 downregulation in cells reprogrammed with HNF1B R295C, suggesting that our model might be suitable to untangle the genetic regulatory circuits of HIF-1ɑ stabilization from metabolic or other signals mimicking hypoxia and contributing to cyst development. Such network perturbations will require follow-up analysis in animal models and patient samples.

It has been postulated that HNF1B acts in renal repair mechanisms, which potentially can be inflicted by hypoxic damage. Hnf1b is initially downregulated in models of acute tubular injury, permitting the derepression of SOCS3.^112^ We also find SOCS3 upregulated in the reprogramming conditions with pathological HNF1B, suggesting that HNF1B R295C can partially activate tubular repair mechanisms.

HNF1B mutations can not only lead to cystic dysplastic kidneys but also elicit more severe manifestations of CAKUT, including renal agenesis. One of the Hnf1b targets is Lama5, an extracellular matrix component and a major constituent of basement membranes. While being vital for kidney glomerular development and function,^113^ mutant mice can also lack one or both kidneys completely.^114^ Mutations in *LAMA5* have been detected in one patient with CAKUT leading to its classification as a disease candidate gene.^5^ We also noticed that iRECs reprogrammed with HNF1B R295C were more adherent and showed an altered morphology in comparison with iREC generated with WT HNF1B (Supplemental Figure 6B). Interestingly, a similar phenotype has been described in Hnf1b knockout mIMCD3 cells.^115^ Consistent with a repressive effect of Hnf1b on the profibrotic transcription factor Twist2, we likewise see upregulation of Twist2 in cells reprogrammed with HNF1B R295C. These findings suggest a potential role for ECM and cell adherence remodeling in HNF1B-associated kidney disease and will need to be complemented by analysis of patient samples, iPSC-derived organoids, or in other animal models.

In conclusion, we show that it is possible to generate renal-like organoids from *Xenopus* ectodermal explants by expressing the transcription factor Hnf1a, Hnf1b, or Sall1. We also demonstrate that inserting patient-specific mutations in direct reprogramming factors can convert fibroblasts to renal epithelial cells that allow detailed analysis of transcriptional changes. The combinatorial use of two *de novo* nephrogenic models in two vertebrate species constitutes a novel approach to uncover evolutionarily conserved gene programs that control kidney development and may be dysregulated in genetic disease. Our findings add important insights into the complex regulatory function of HNF1B in renal development and maintenance and provide a rich resource of novel functional HNF1B targets to explore in future studies.

## Supplementary Material

## Data Availability

The FASTQ sequences reported in this article have been deposited in the NCBI Sequence Read Archive under BioProject PRJNA727068. All data are available from the authors upon request.

## Supplemental Material

This article contains the following supplemental material online at http://links.lww.com/JSN/D602, http://links.lww.com/JSN/D603, http://links.lww.com/JSN/D629, http://links.lww.com/JSN/D636, http://links.lww.com/JSN/D996.

Supplemental Figure 1. Inducing renal tissue with HNF1B patient-specific mutation R295C.

Supplemental Figure 2. RNA sequencing of the induced renal tissue.

Supplemental Figure 3. Transcriptional alterations due to HNF1B patient-specific mutation R295C.

Supplemental Figure 4. Transcriptional stability of the iRECs and genes essential for kidney development in *Xenopus* organoids.

Supplemental Figure 5. *In vivo* analysis of *hnf1b* and candidate genes.

Supplemental Figure 6. Analysis of HNF1B targets in iRECs and *in vivo*.

Supplemental Table 1. DESeq2 results from mouse RNA sequencing.

Supplemental Table 2. DESeq2 results from Xenopus explant RNA sequencing.

Supplemental Table 3. Cluster-specific genes.

Supplemental Table 4. Genes contributing to the GO terms in Figure 3D.

Supplemental Table 5. Signal in the pronephric area for the 36 *in vivo* validated candidate genes.

Supplemental Table 6. Primers used for mutation cloning.

Supplemental Table 7. Primers used for 36 targets cloning.

Supplemental Table 8. Sequences of the sgRNAs and MO from microinjection experiments.

Supplemental Table 9. Antibodies and dilutions.

Supplemental Table 10. Primers used for qPCR.

## References

[1] Choi HA Lee DJ Shin SM Lee YK Ko SY Park SW. The prenatal and postnatal incidence of congenital anomalies of the kidneys and urinary tract (CAKUT) detected by ultrasound. Child Kidney Dis. 2016;20(1):29-32. doi:10.3339/jkspn.2016.20.1.29

[2] Andrés-Jensen L Jørgensen FS Thorup J . The outcome of antenatal ultrasound diagnosed anomalies of the kidney and urinary tract in a large Danish birth cohort. Arch Dis Child. 2016;101(9):819-824. doi:10.1136/archdischild-2015-309784 27217581

[3] Laurichesse Delmas H Kohler M Doray B . Congenital unilateral renal agenesis: prevalence, prenatal diagnosis, associated anomalies. Data from two birth-defect registries. Birth Defects Res. 2017;109(15):1204-1211. doi:10.1002/bdr2.1065 28722320

[4] Seikaly MG Ho PL Emmett L Fine RN Tejani A. Chronic renal insufficiency in children: The 2001 Annual Report of the NAPRTCS. Pediatr Nephrol. 2003;18(8):796-804. doi:10.1007/s00467-003-1158-5 12811650

[5] van der Ven AT Connaughton DM Ityel H . Whole-exome sequencing identifies causative mutations in families with congenital anomalies of the kidney and urinary tract. J Am Soc Nephrol. 2018;29(9):2348-2361. doi:10.1681/ASN.2017121265 30143558 PMC6115658

[6] Heidet L Decramer S Pawtowski A . Spectrum of HNF1B mutations in a large cohort of patients who harbor renal diseases. Clin J Am Soc Nephrol. 2010;5(6):1079-1090. doi:10.2215/CJN.06810909 20378641 PMC2879303

[7] Nakayama M Nozu K Goto Y . HNF1B alterations associated with congenital anomalies of the kidney and urinary tract. Pediatr Nephrol. 2010;25(6):1073-1079. doi:10.1007/s00467-010-1454-9 20155289

[8] Ulinski T Lescure S Beaufils S . Renal phenotypes related to hepatocyte nuclear factor-1β (*TCF2*) mutations in a pediatric cohort. J Am Soc Nephrol. 2006;17(2):497-503. doi:10.1681/ASN.2005101040 16371430

[9] Alvelos MI Rodrigues M Lobo L . A novel mutation of the HNF1B gene associated with hypoplastic glomerulocystic kidney disease and neonatal renal failure: a case report and mutation update. Medicine (Baltimore). 2015;94(7):e469. doi:10.1097/md.0000000000000469 25700310 PMC4554182

[10] Clissold RL Ashfield B Burrage J . Genome-wide methylomic analysis in individuals with HNF1B intragenic mutation and 17q12 microdeletion. Clin Epigenetics. 2018;1097(1):97. doi:10.1186/s13148-018-0530-z PMC6052548 30021660

[11] Bingham C Bulman MP Ellard S . Mutations in the hepatocyte nuclear factor-1β gene are associated with familial hypoplastic glomerulocystic kidney disease. Am J Hum Genet. 2001;68(1):219-224. doi:10.1086/316945 11085914 PMC1234916

[12] Thomas R Sanna-Cherchi S Warady BA Furth SL Kaskel FJ Gharavi AG. HNF1B and PAX2 mutations are a common cause of renal hypodysplasia in the CKiD cohort. Pediatr Nephrol. 2011;26(6):897-903. doi:10.1007/s00467-011-1826-9 21380624 PMC3257470

[13] Weber S Moriniere V Knüppel T . Prevalence of mutations in renal developmental genes in children with renal hypodysplasia: Results of the ESCAPE study. J Am Soc Nephrol. 2006;17(10):2864-2870. doi:10.1681/ASN.2006030277 16971658

[14] Wang L Coffinier C Thomas MK . Selective deletion of the Hnf1β (MODY5) gene in β-cells leads to altered gene expression and defective insulin release. Endocrinology. 2004;145(8):3941-3949. doi:10.1210/en.2004-0281 15142986

[15] Horikawa Y Iwasaki N Hara M . Mutation in hepatocyte nuclear factor-1 beta gene (TCF2) associated with MODY. Nat Genet. 1997;17(4):384-385. doi:10.1038/ng1297-384 9398836

[16] Strazzabosco M Fabris L. Development of the bile ducts: essentials for the clinical hepatologist. J Hepatol. 2012;56(5):1159-1170. doi:10.1016/j.jhep.2011.09.022 22245898 PMC3328609

[17] Limaye PB Alarcón G Walls AL . Expression of specific hepatocyte and cholangiocyte transcription factors in human liver disease and embryonic development. Lab Invest. 2008;88(8):865-872. doi:10.1038/labinvest.2008.56 18574450 PMC2631390

[18] Clissold RL Hamilton AJ Hattersley AT Ellard S Bingham C. HNF1B-associated renal and extra-renal disease—an expanding clinical spectrum. Nat Rev Nephrol. 2015;11(2):102-112. doi:10.1038/nrneph.2014.232 25536396

[19] Stanley KE Giordano J Thorsten V . Causal genetic variants in stillbirth. N Engl J Med. 2020;383(12):1107-1116. doi:10.1056/nejmoa1908753 32786180 PMC7604888

[20] Karczewski KJ Francioli LC Tiao G ; Genome Aggregation Database Consortium. The mutational constraint spectrum quantified from variation in 141,456 humans. Nature. 2020;581(7809):434-443. doi:10.1038/s41586-020-2308-7 32461654 PMC7334197

[21] Hiesberger T Shao X Gourley E Reimann A Pontoglio M Igarashi P. Role of the hepatocyte nuclear factor-1β (HNF-1β) C-terminal domain in Pkhd1 (ARPKD) gene transcription and renal cystogenesis. J Biol Chem. 2005;280(11):10578-10586. doi:10.1074/jbc.m414121200 15647252

[22] Barbacci E Chalkiadaki A Masdeu C . HNF1 β/TCF2 mutations impair transactivation potential through altered co-regulator recruitment. Hum Mol Genet. 2004;13(24):3139-3149. doi:10.1093/hmg/ddh338 15509593

[23] Cereghini S. Liver-enriched transcription factors and hepatocyte differentiation. FASEB J. 1996;10(2):267-282. doi:10.1096/fasebj.10.2.8641560 8641560

[24] Ferrè S de Baaij JHF Ferreira P . Mutations in PCBD1 cause hypomagnesemia and renal magnesium wasting. J Am Soc Nephrol. 2014;25(3):574-586. doi:10.1681/ASN.2013040337 24204001 PMC3935582

[25] Teo AKK Lau HH Valdez IA . Early developmental perturbations in a human stem cell model of MODY5/HNF1B pancreatic hypoplasia. Stem Cell Rep. 2016;6(3):357-367. doi:10.1016/j.stemcr.2016.01.007 PMC4788763 26876668

[26] Wang J He C Gao P . HNF1B-mediated repression of SLUG is suppressed by EZH2 in aggressive prostate cancer. Oncogene. 2020;39(6):1335-1346. doi:10.1038/s41388-019-1065-2 31636385 PMC7002300

[27] Lu W Sun J Zhou H . HNF1B inhibits cell proliferation via repression of SMAD6 expression in prostate cancer. J Cell Mol Med. 2020;24:14539-14548. doi:10.1111/jcmm.16081 33174391 PMC7754016

[28] Ferrè S Veenstra GJC Bouwmeester R Hoenderop JGJ Bindels RJM. HNF-1B specifically regulates the transcription of the γa-subunit of the Na+/K+-ATPase. Biochem Biophysical Res Commun. 2011;404(1):284-290. doi:10.1016/j.bbrc.2010.11.108 21130072

[29] Lu P Rha GB Chi Y-I. Structural basis of disease-causing mutations in hepatocyte nuclear factor 1β. Biochemistry. 2007;46(43):12071-12080. doi:10.1021/bi7010527 17924661 PMC2367142

[30] Ceska TA Lamers M Monaci P Nicosia A Cortese R Suck D. The X-ray structure of an atypical homeodomain present in the rat liver transcription factor LFB1/HNF1 and implications for DNA binding. EMBO J. 1993;12(5):1805-1810. doi:10.1002/j.1460-2075.1993.tb05828.x 8491173 PMC413399

[31] Bohn S Thomas H Turan G . Distinct molecular and morphogenetic properties of mutations in the human HNF1β gene that lead to defective kidney development. J Am Soc Nephrol. 2003;14(8):2033-2041. doi:10.1097/01.ASN.0000078808.70309.c4 12874457

[32] Lamb TM Knecht AK Smith WC . Neural induction by the secreted polypeptide noggin. Science. 1993;262(5134):713-718. doi:10.1126/science.8235591 8235591

[33] Niborski LL Paces-Fessy M Ricci P . Hnf1b haploinsufficiency differentially affects developmental target genes in a new renal cysts and diabetes mouse model. Dis Models Mech. 2021;14(5):dmm047498. doi:10.1242/dmm.047498 PMC8126479 33737325

[34] Lokmane L Heliot C Garcia-Villalba P Fabre M Cereghini S. vHNF1 functions in distinct regulatory circuits to control ureteric bud branching and early nephrogenesis. Development. 2010;137(2):347-357. doi:10.1242/dev.042226 20040500

[35] Barbacci E Reber M Ott MO Breillat C Huetz F Cereghini S. Variant hepatocyte nuclear factor 1 is required for visceral endoderm specification. Development. 1999;126(21):4795-4805. doi:10.1242/dev.126.21.4795 10518496

[36] Faguer S Decramer S Chassaing N . Diagnosis, management, and prognosis of HNF1B nephropathy in adulthood. Kidney Int. 2011;80(7):768-776. doi:10.1038/ki.2011.225 21775974

[37] Eckardt K-U Alper SL Antignac C . Autosomal dominant tubulointerstitial kidney disease: diagnosis, classification, and management—a KDIGO consensus report. Kidney Int. 2015;88(4):676-683. doi:10.1038/ki.2015.28 25738250

[38] Adalat S Woolf AS Johnstone KA . HNF1B mutations associate with hypomagnesemia and renal magnesium wasting. J Am Soc Nephrol. 202009;20(5):1123-1131. doi:10.1681/ASN.2008060633 PMC2678044 19389850

[39] Kaminski MM Tosic J Kresbach C . Direct reprogramming of fibroblasts into renal tubular epithelial cells by defined transcription factors. Nat Cell Biol. 2016;18(12):1269-1280. doi:10.1038/ncb3437 27820600

[40] Marchesin V Pérez-Martí A Le Meur G . Molecular basis for autosomal-dominant renal fanconi syndrome caused by HNF4A. Cell Rep. 2019;29(13):4407-4421.e5. doi:10.1016/j.celrep.2019.11.066 31875549 PMC6941224

[41] Moriya N Uchiyama H Asashima M. Induction of pronephric tubules by activin and retinoic acid in presumptive ectoderm of Xenopus laevis. (RA/kidney/mesoderm induction/Xenopus laevis). Dev Growth Differ. 1993;35(2):123-128. doi:10.1111/j.1440-169x.1993.00123.x 37281298

[42] Getwan M Lienkamp SS. Toolbox in a tadpole: Xenopus for kidney research. Cell Tissue Res. 2017;369(1):143-157. doi:10.1007/s00441-017-2611-2 28401306

[43] Lienkamp SS. Using Xenopus to study genetic kidney diseases. Semin Cell Develop Biol. 2016;51:117-124. doi:10.1016/j.semcdb.2016.02.002 26851624

[44] Lienkamp SS Liu K Karner CM . Vertebrate kidney tubules elongate using a planar cell polarity-dependent, rosette-based mechanism of convergent extension. Nat Genet. 2012;44(12):1382-1387. doi:10.1038/ng.2452 23143599 PMC4167614

[45] Raciti D Reggiani L Geffers L . Organization of the pronephric kidney revealed by large-scale gene expression mapping. Genome Biol. 2008;9(5):R84. doi:10.1186/gb-2008-9-5-r84 18492243 PMC2441470

[46] Zhou X Vize PD. Proximo-distal specialization of epithelial transport processes within the Xenopus pronephric kidney tubules. Develop Biol. 2004;271(2):322-338. doi:10.1016/j.ydbio.2004.03.036 15223337

[47] Hoff S Halbritter J Epting D . ANKS6 is a central component of a nephronophthisis module linking NEK8 to INVS and NPHP3. Nat Genet. 2013;45(8):951-956. doi:10.1038/ng.2681 23793029 PMC3786259

[48] Lienkamp S Ganner A Boehlke C . Inversin relays Frizzled-8 signals to promote proximal pronephros development. Proc Natl Acad Sci U S A. 2010;107(47):20388-20393. doi:10.1073/pnas.1013070107 21059920 PMC2996658

[49] Faber J Nieuwkoop PD: *Normal table ofxenopus laevis (Daudin)*, New York, Garland Publishing Inc, 1994.

[50] Nakayama T Fish MB Fisher M Oomen-Hajagos J Thomsen GH Grainger RM. Simple and efficient CRISPR/Cas9-mediated targeted mutagenesis in Xenopus tropicalis. Genesis. 2013;51(12):835-843. doi:10.1002/dvg.22720 24123613 PMC3947545

[51] Schindelin J Arganda-Carreras I Frise E . Fiji: an open-source platform for biological-image analysis. Nat Methods. 2012;9(7):676-682. doi:10.1038/nmeth.2019 22743772 PMC3855844

[52] Falk T Mai D Bensch R . U-Net: deep learning for cell counting, detection, and morphometry. Nat Methods. 2019;16(1):67-70. doi:10.1038/s41592-018-0261-2 30559429

[53] Naert T Çiçek Ö Ogar P . Deep learning is widely applicable to phenotyping embryonic development and disease. Development. 2021;148(21):dev199664. doi:10.1242/dev.199664 34739029 PMC8602947

[54] Voigt FF Kirschenbaum D Platonova E . The mesoSPIM initiative: open-source light-sheet microscopes for imaging cleared tissue. Nat Methods. 2019;16(11):1105-1108. doi:10.1038/s41592-019-0554-0 31527839 PMC6824906

[55] Sive HL Grainger RM Harland RM. Early Development of Xenopus laevis: A Laboratory Manual. Cold Spring Harbor Laboratory Press.

[56] Tran U Pickney LM Ozpolat BD Wessely O. Xenopus Bicaudal-C is required for the differentiation of the amphibian pronephros. Develop Biol. 2007;307(1):152-164. doi:10.1016/j.ydbio.2007.04.030 17521625 PMC1976305

[57] Schmittgen TD Livak KJ. Analyzing real-time PCR data by the comparative CT method. Nat Protoc. 2008;3(6):1101-1108. doi:10.1038/nprot.2008.73 18546601

[58] Afgan E Baker D Batut B . The Galaxy platform for accessible, reproducible and collaborative biomedical analyses: 2018 update. Nucleic Acids Res. 2018;46(W1):W537–W544. doi:10.1093/nar/gky379 29790989 PMC6030816

[59] Dobin A Davis CA Schlesinger F . STAR: ultrafast universal RNA-seq aligner. Bioinformatics. 2013;29(1):15-21. doi:10.1093/bioinformatics/bts635 23104886 PMC3530905

[60] Liao Y Smyth GK Shi W. featureCounts: an efficient general purpose program for assigning sequence reads to genomic features. Bioinformatics. 2014;30(7):923-930. doi:10.1093/bioinformatics/btt656 24227677

[61] Love MI Huber W Anders S. Moderated estimation of fold change and dispersion for RNA-seq data with DESeq2. Genome Biol. 2014;15(12):550. doi:10.1186/s13059-014-0550-8 25516281 PMC4302049

[62] Blighe K Lun A. PCAtools: Everything Principal Components Analysis. R Package Version 2.2.0; 2020. https://github.com/kevinblighe/PCAtools. Accessed April 23, 2021

[63] Kumar L Futschik ME. Mfuzz: a software package for soft clustering of microarray data. Bioinformation. 2007;2(1):5-7. doi:10.6026/97320630002005 18084642 PMC2139991

[64] Gentleman RC Carey VJ Bates DM . Bioconductor: open software development for computational biology and bioinformatics. Genome Biol. 2004;5(10):R80. doi:10.1186/gb-2004-5-10-r80 15461798 PMC545600

[65] Huber W Carey VJ Gentleman R . Orchestrating high-throughput genomic analysis with Bioconductor. Nat Methods. 2015;12(2):115-121. doi:10.1038/nmeth.3252 25633503 PMC4509590

[66] Yu G Wang L-G Han Y He Q-Y. clusterProfiler: an R Package for comparing biological themes among gene clusters. OMICS. 2012;16(5):284-287. doi:10.1089/omi.2011.0118 22455463 PMC3339379

[67] Wickham H. ggplot2: Elegant Graphics for Data Analysis. Springer-Verlag. https://ggplot2.tidyverse.org. Accessed March 18, 2021

[68] Warnes GR Bolker B Bonebakker L gplots: Various R Programming Tools for Plotting Data. R Package Version 2.17.0; 2015. http://CRAN.R-project.org/package=gplots. Accessed March 18, 2021

[69] Gu Z Eils R Schlesner M. Complex heatmaps reveal patterns and correlations in multidimensional genomic data. Bioinformatics. 2016;32(18):2847-2849. doi:10.1093/bioinformatics/btw313 27207943

[70] Chen H Boutros PC. VennDiagram: a package for the generation of highly-customizable Venn and Euler diagrams in R. BMC Bioinformatics. 2011;12(1):35. doi:10.1186/1471-2105-12-35 21269502 PMC3041657

[71] Blighe K Rana S Lewis M: EnhancedVolcano: publication-ready volcano plots with enhanced colouring and labeling. R package version 1.8.0. 2020. https://github.com/kevinblighe/EnhancedVolcano. Accessed April 23, 2021

[72] Harrell F. Hmisc. https://github.com/harrelfe/Hmisc/. Accessed March 18, 2021

[73] Wickham H Averick M Bryan J . Welcome to the Tidyverse. J Open Source Softw. 2019;4(43):1686. doi:10.21105/joss.01686

[74] Wickham H. Reshaping data with the reshape package. J Stat Softw. 2007;21(12):1-20. doi:10.18637/jss.v021.i12

[75] Drost H-G Paszkowski J. Biomartr: genomic data retrieval with R. Bioinformatics. 2017;33(8):1216-1217. doi:10.1093/bioinformatics/btw821 28110292 PMC5408848

[76] Karimi K Fortriede JD Lotay VS . Xenbase: a genomic, epigenomic and transcriptomic model organism database. Nucleic Acids Res. 2018;46(D1):D861–D868. doi:10.1093/nar/gkx936 29059324 PMC5753396

[77] GTEx Consortium. The Genotype-Tissue Expression (GTEx) project. Nat Genet. 2013;45(6):580-585. doi:10.1038/ng.2653 23715323 PMC4010069

[78] Oki S Ohta T Shioi G ChIP‐Atlas: a data‐mining suite powered by full integration of public ChIP‐seq data. EMBO Rep. 2018;19:e46255. doi:10.15252/embr.201846255 30413482 PMC6280645

[79] Pontén F Jirström K Uhlen M. The Human Protein Atlas—a tool for pathology. J Pathol. 2008;216(4):387-393. doi:10.1002/path.2440 18853439

[80] Bellanné-Chantelot C Chauveau D Gautier J-F . Clinical spectrum associated with hepatocyte nuclear factor-1β mutations. Ann Intern Med. 2004;140(7):510-517. doi:10.7326/0003-4819-140-7-200404060-00009 15068978

[81] Bellanné-Chantelot C Clauin S Chauveau D . Large genomic rearrangements in the hepatocyte nuclear factor-1β (*TCF2*) gene are the most frequent cause of maturity-onset diabetes of the young type 5. Diabetes. 2005;54(11):3126-3132. doi:10.2337/diabetes.54.11.3126 16249435

[82] Kumaran GK Hanukoglu I. Identification and classification of epithelial cells in nephron segments by actin cytoskeleton patterns. FEBS J. 2019;287(6):1176-1194. doi:10.1111/febs.15088 31605441 PMC7384063

[83] Heliot C Desgrange A Buisson I . HNF1B controls proximal-intermediate nephron segment identity in vertebrates by regulating Notch signalling components and Irx1/2. Development. 2013;140(4):873-885. doi:10.1242/dev.086538 23362348

[84] Sander V Salleh L Naylor RW . Transcriptional profiling of the zebrafish proximal tubule. Am J Physiol Renal Physiol. 2019;317(2):F478–F488. doi:10.1152/ajprenal.00174.2019 31188030

[85] Desgrange A Heliot C Skovorodkin I . HNF1B controls epithelial organization and cell polarity during ureteric bud branching and collecting duct morphogenesis. Development. 2017;144(24):4704-4719. doi:10.1242/dev.154336 29158444

[86] Roelandt P Antoniou A Libbrecht L . HNF1B deficiency causes ciliary defects in human cholangiocytes. Hepatology. 2012;56(3):1178-1181. doi:10.1002/hep.25876 22706971

[87] Bergmann C. Educational paper: ciliopathies. Eur J Pediatr. 2012;171(9):1285-1300. doi:10.1007/s00431-011-1553-z 21898032 PMC3419833

[88] Hildebrandt F Benzing T Katsanis N. Ciliopathies. N Engl J Med. 2011;364(16):1533-1543. doi:10.1056/nejmra1010172 21506742 PMC3640822

[89] Lu H Galeano MCR Ott E . Mutations in DZIP1L, which encodes a ciliary-transition-zone protein, cause autosomal recessive polycystic kidney disease. Nat Genet. 2017;49(7):1025-1034. doi:10.1038/ng.3871 28530676 PMC5687889

[90] Nigam A Knoers NVAM Renkema KY. Impact of next generation sequencing on our understanding of CAKUT. Semin Cell Dev Biol. 2019;91:104-110. doi:10.1016/j.semcdb.2018.08.013 30172048

[91] The European Polycystic Kidney Disease Consortium. The polycystic kidney disease 1 gene encodes a 14 kb transcript and lies within a duplicated region on chromosome 16. Cell. 1994;77(6):881-894. doi:10.1016/0092-8674(94)90137-6 8004675

[92] Mochizuki T Wu G Hayashi T . *PKD2*, a gene for polycystic kidney disease that encodes an integral membrane protein. Science. 1996;272(5266):1339-1342. doi:10.1126/science.272.5266.1339 8650545

[93] Shen Y Yue F McCleary DF . A map of the cis-regulatory sequences in the mouse genome. Nature. 2012;488(7409):116-120. doi:10.1038/nature11243 22763441 PMC4041622

[94] Decramer S Parant O Beaufils S . Anomalies of the TCF2 gene are the main cause of fetal bilateral hyperechogenic kidneys. J Am Soc Nephrol. 2007;18(3):923-933. doi:10.1681/ASN.2006091057 17267738

[95] Aboudehen K Kim MS Mitsche M . Transcription factor hepatocyte nuclear factor-1β regulates renal cholesterol metabolism. J Am Soc Nephrol. 2016;27(8):2408-2421. doi:10.1681/ASN.2015060607 26712526 PMC4978044

[96] Kompatscher A de Baaij JHF Aboudehen K . Loss of transcriptional activation of the potassium channel Kir5.1 by HNF1β drives autosomal dominant tubulointerstitial kidney disease. Kidney Int. 2017;92(5):1145-1156. doi:10.1016/j.kint.2017.03.034 28577853 PMC5903269

[97] Drews C Senkel S Ryffel GU. The nephrogenic potential of the transcription factors osr1, osr2, hnf1b, lhx1 and pax8 assessed in Xenopus animal caps. BMC Dev Biol. 2011;11:5. doi:10.1186/1471-213x-11-5 21281489 PMC3042965

[98] Ferrè S Igarashi P. New insights into the role of HNF-1β in kidney (patho)physiology. Pediatr Nephrol. 2019;34(8):1325-1335. doi:10.1007/s00467-018-3990-7 29961928 PMC6312759

[99] Gresh L Fischer E Reimann A . A transcriptional network in polycystic kidney disease. EMBO J. 2004;23(7):1657-1668. doi:10.1038/sj.emboj.7600160 15029248 PMC391068

[100] Hiesberger T Bai Y Shao X . Mutation of hepatocyte nuclear factor–1β inhibits Pkhd1 gene expression and produces renal cysts in mice. J Clin Invest. 2004;113(6):814-825. doi:10.1172/jci200420083 15067314 PMC362119

[101] Hildebrandt F Attanasio M Otto E. Nephronophthisis: disease mechanisms of a ciliopathy. J Am Soc Nephrol. 202009;20:23-35. doi:10.1681/ASN.2008050456 PMC2807379 19118152

[102] Attanasio M Uhlenhaut NH Sousa VH . Loss of GLIS2 causes nephronophthisis in humans and mice by increased apoptosis and fibrosis. Nat Genet. 2007;39(8):1018-1024. doi:10.1038/ng2072 17618285

[103] Zaucke F Boehnlein JM Steffens S . Uromodulin is expressed in renal primary cilia and UMOD mutations result in decreased ciliary uromodulin expression. Hum Mol Genet. 2010;19(10):1985-1997. doi:10.1093/hmg/ddq077 20172860 PMC2860893

[104] Yoder BK Hou X Guay-Woodford LM. The polycystic kidney disease proteins, polycystin-1, polycystin-2, polaris, and cystin, are co-localized in renal cilia. J Am Soc Nephrol. 2002;13(10):2508-2516. doi:10.1097/01.ASN.0000029587.47950.25 12239239

[105] Lea WA Ward CJ. A new epitope-tagged Pkhd1 allele sheds light on fibrocystin signaling. Kidney Int. 2017;92(5):1041-1043. doi:10.1016/j.kint.2017.05.026 29055424

[106] Aboudehen K Noureddine L Cobo-Stark P . Hepatocyte nuclear factor-1β regulates urinary concentration and response to hypertonicity. J Am Soc Nephrol. 2017;28(10):2887-2900. doi:10.1681/ASN.2016101095 28507058 PMC5619957

[107] Robinson CM Poon BPK Kano Y Pluthero FG Kahr WHA Ohh M. A hypoxia-inducible HIF1–GAL3ST1-sulfatide Axis enhances ccRCC immune evasion via increased tumor cell–platelet binding. Mol Cancer Res. 2019;17(11):2306-2314. doi:10.1158/1541-7786.mcr-19-0461 31427440

[108] Stettner P Bourgeois S Marsching C . Sulfatides are required for renal adaptation to chronic metabolic acidosis. Proc Natl Acad Sci U S A. 2013;110(24):9998-10003. doi:10.1073/pnas.1217775110 23716689 PMC3683795

[109] Tanaka T Miyata T Inagi R Fujita T Nangaku M. Hypoxia in renal disease with proteinuria and/or glomerular hypertension. Am J Pathol. 2004;165(6):1979-1992. doi:10.1016/s0002-9440(10)63249-x 15579441 PMC1618699

[110] Buchholz B Schley G Faria D . Hypoxia-inducible factor-1α causes renal cyst expansion through calcium-activated chloride secretion. J Am Soc Nephrol. 2014;25(3):465-474. doi:10.1681/ASN.2013030209 24203996 PMC3935579

[111] Pollard PJ Spencer-Dene B Shukla D . Targeted inactivation of fh1 causes proliferative renal cyst development and activation of the hypoxia pathway. Cancer Cell. 2007;11(4):311-319. doi:10.1016/j.ccr.2007.02.005 17418408

[112] Faguer S Mayeur N Casemayou A . Hnf-1β transcription factor is an early hif-1α-independent marker of epithelial hypoxia and controls renal repair. PLoS One. 2013;8(5):e63585. doi:10.1371/journal.pone.0063585 23704921 PMC3660442

[113] Steenhard BM Zelenchuk A Stroganova L . Transgenic expression of human LAMA5 suppresses murine Lama5 mRNA and laminin α5 protein deposition. PLoS One. 2011;6(9):e23926. doi:10.1371/journal.pone.0023926 21915268 PMC3168496

[114] Miner JH Li C. Defective glomerulogenesis in the absence of laminin α5 demonstrates a developmental role for the kidney glomerular basement membrane. Develop Biol. 2000;217(2):278-289. doi:10.1006/dbio.1999.9546 10625553

[115] Chan SC Zhang Y Shao A . Mechanism of fibrosis in *HNF1B*-related autosomal dominant tubulointerstitial kidney disease. J Am Soc Nephrol. 2018;29(10):2493-2509. doi:10.1681/ASN.2018040437 30097458 PMC6171276

