# Supplementary material for: HNF1B Alters an Evolutionarily Conserved Nephrogenic Program of Target Genes
Source: J Am Soc Nephrol. 2022 Dec 15;34(3):412–32. doi: 10.1681/ASN.2022010076 (PMC10103355; doi:10.1681/ASN.2022010076)
Supplement: Supplementary file 1 [file jasn-34-412-s001.pdf]

SUPPLEMENTARY INFORMATION

Supplementary Figure 1
Supplementary Figure 2
Supplementary Figure 3
Supplementary Figure 4
Supplementary Figure 5
Supplementary Figure 6
Supplementary Table 1. DESeq2 results from Mouse RNA sequencing
Supplementary Table 2. DESeq2 results from Xenopus explant RNA sequencing
Supplementary Table 3. Cluster specific genes
Supplementary Table 4. Genes contributing to the GO terms in Fig. 3D
Supplementary Table 5. Signal in the pronephric area for the 36 in vivo validated candidate genes
Supplementary Table 6. Primers used for mutation cloning
Supplementary Table 7. Primers used for 36 targets cloning

Supplementary Figure 1

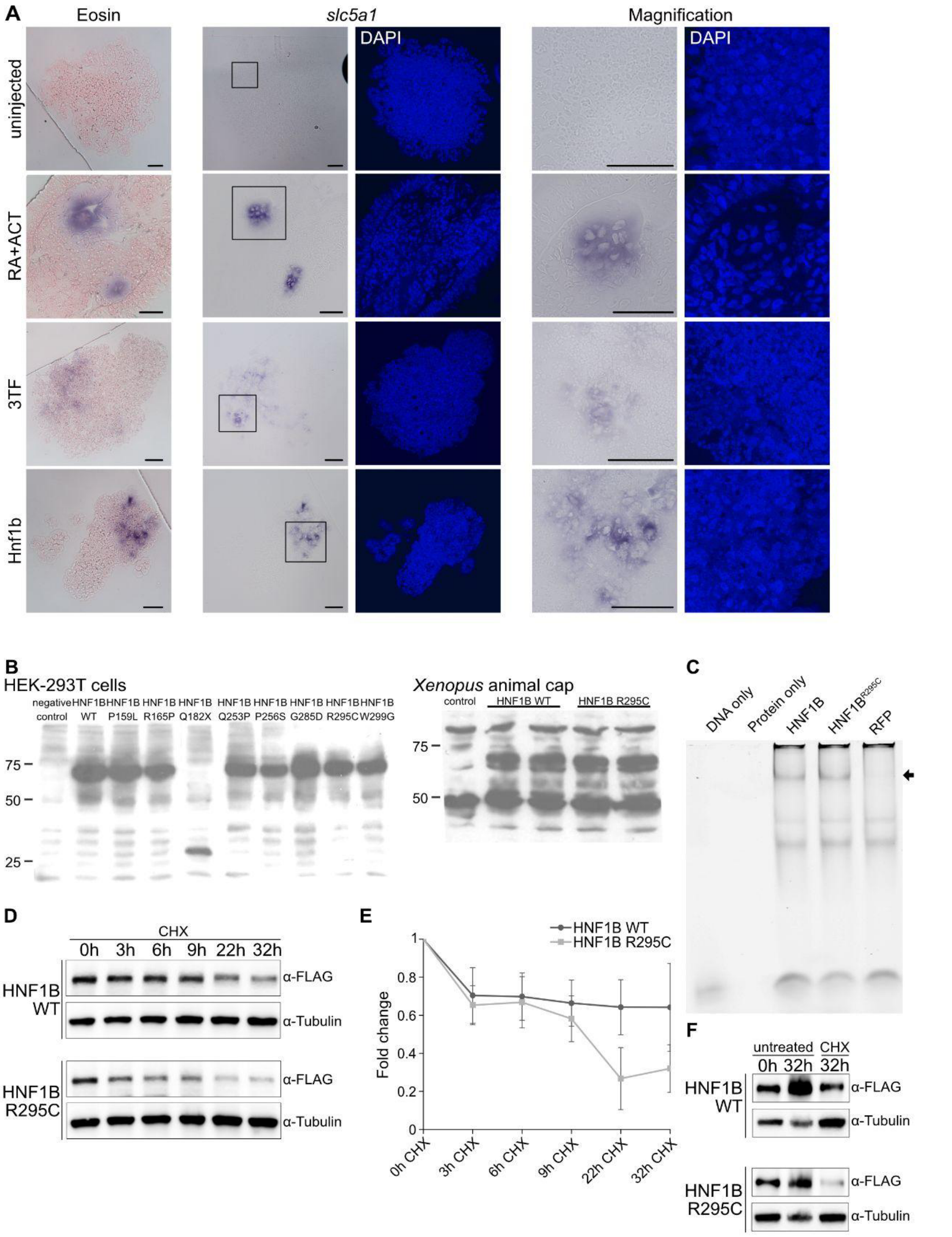

Supplementary Figure 1 related to Figure 1 and 2. **Inducing renal tissue with HNF1B patient-specific mutation R295C**. **A**. Microtome sections of *in situ* hybridisation for *slc5a1 of* animal caps treated with retinoic acid and actin (RA+ACT), extracted from tadpoles co-injected with mRNA of *hnf1a*, *hnf1b* and *sall1* transcription factors (3TF) or injected with *hnf1b* mRNA. Sections were stained with Eosin and DAPI. All scale bars are 50µm. **B**. Western blot analysis of eight HNF1B mutations expressed in 293T cells (top/left). For the *Xenopus* animal cap induction experiments HNF1B WT or R295C proteins were detected (bottom/right). **C**. DNA binding assay of HNF1B and HNF1B R295C. The lower band indicates free, unbound DNA. The arrow indicates DNA bound to HNF1B proteins. Proteins were purified from transiently transfected HEK293 cells by immunoprecipitation. DNA sequence of the HNF1B binding motif was labelled with Cy7 which was used for detection. **D**. Protein stability of HNF1B WT and HNF1B R259C mutant. Changes in protein expression of FLAG-tagged WT and mutant HNF1B upon Cycloheximide (300µg/ul) treatment for indicated lengths of time (3-32h). **E**. Quantified protein expression of HNF1B WT and HNF1B R295C normalized on Tubulin expression (±SD of minimum n = 3 independent experiments). **F.** Verifying Cycloheximide activity by analyzing protein expression after 32h of treatment.

Supplementary Figure 2

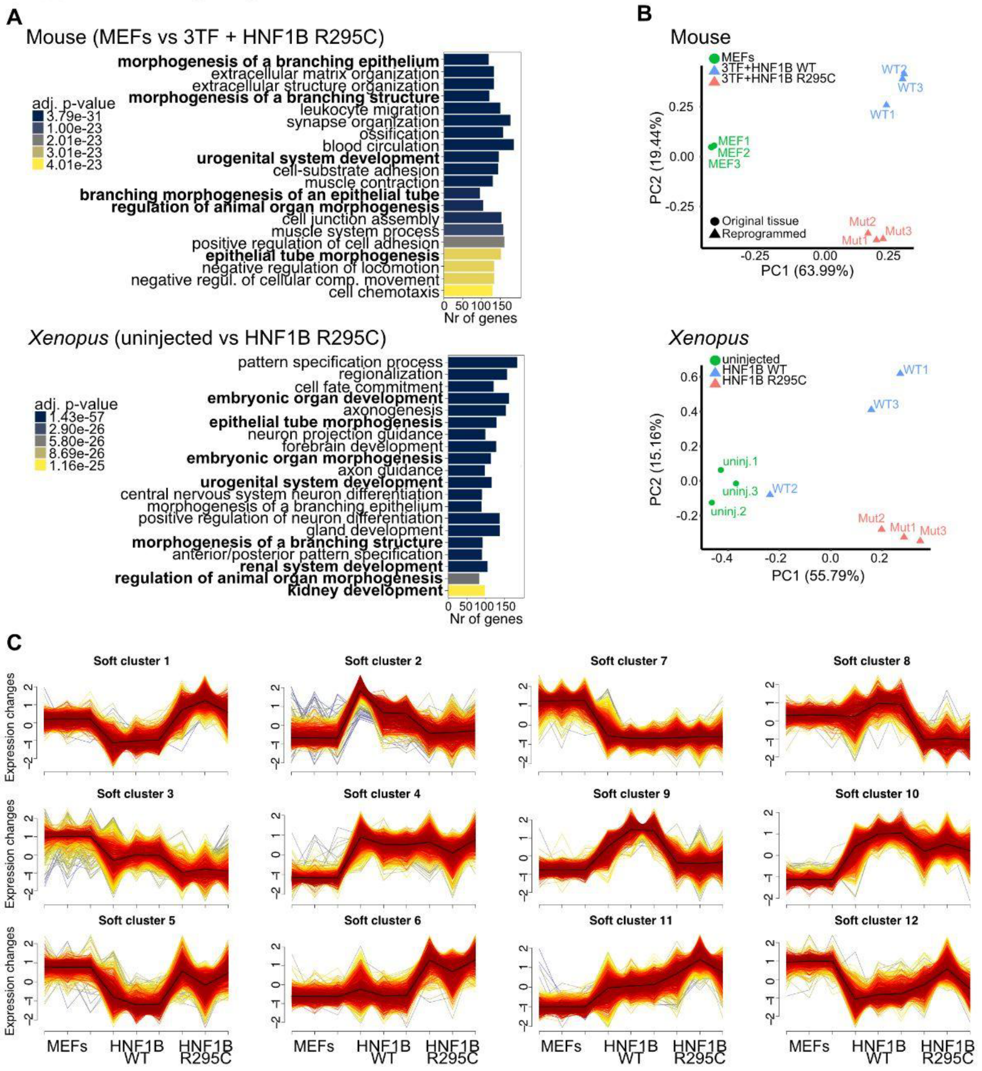

Supplementary Figure 2 related to Figure 2. **RNA sequencing of the induced renal tissue. A**. First two components from principal component analysis in mouse (left) and *Xenopus* (right) experiments. Original tissue is marked with the round shape and reprogrammed tissue with a triangle. Colors indicate the sample type - original tissue (green), reprogramming with HNF1B WT (blue) and reprogramming with HNF1B R295C (red). **B**. Gene Ontology (GO) over-representation test on differentially expressed genes (absolute $\log_2$ fold change >1.5) from the reprogramming process comparing 3TF+ HNF1B R295C iRECs (left) and HNF1B R295C pronephric tissue (right) with original material. GO terms related to renal organogenesis are marked in bold. **C**. Mfuzz soft clustering results from the mouse experiments. Each line represents the expression change between original tissue, 3TF+HNF1B WT and 3TF+HNF1B R295C conditions. Yellow colors indicate low and red high membership values (the similarity of vectors to each other).

Supplementary Figure 3

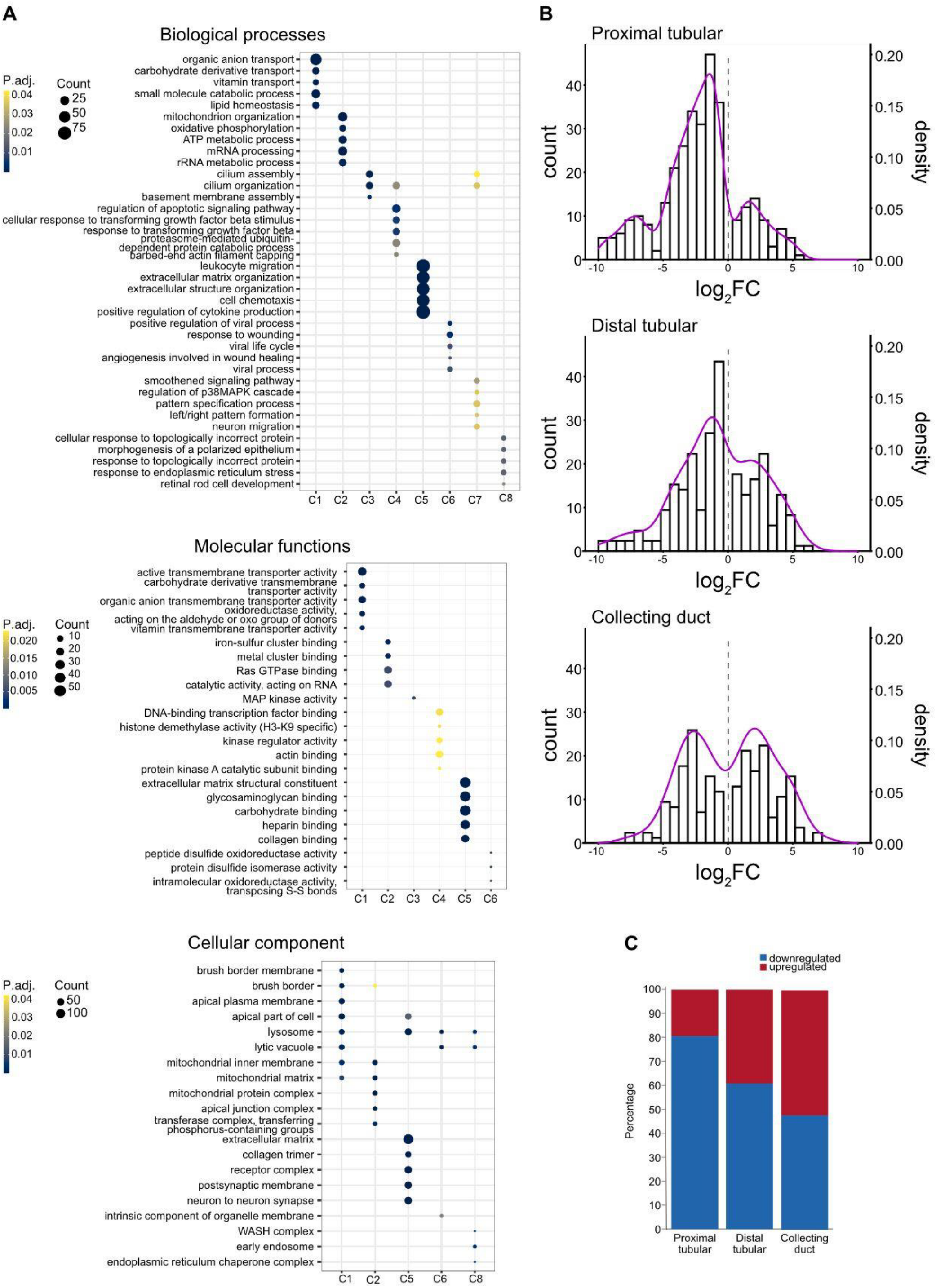

Supplementary Figure 3 related to Figure 3. **Transcriptional alterations due to HNF1B patient-specific mutation R295C**. **A**. Full plot of comparing clusters on biological processes, cellular component and molecular function GO levels. **B**. Distribution of expression changes between 3TF + HNF1B R295C vs 3TF + HNF1B WT in enhanced genes in collecting duct, distal tubular and proximal tubular cells using the single-cell datasets from the Human Protein atlas. Histograms show the genes count (left y-axis) per $\log_2$ fold change (FC) and density plot (purple) illustrating distribution of the plot using kernel smoothing (right y-axis). **C**. The percentage of genes up- (red) or downregulated (blue) in the different renal cell types.

Supplementary Figure 4

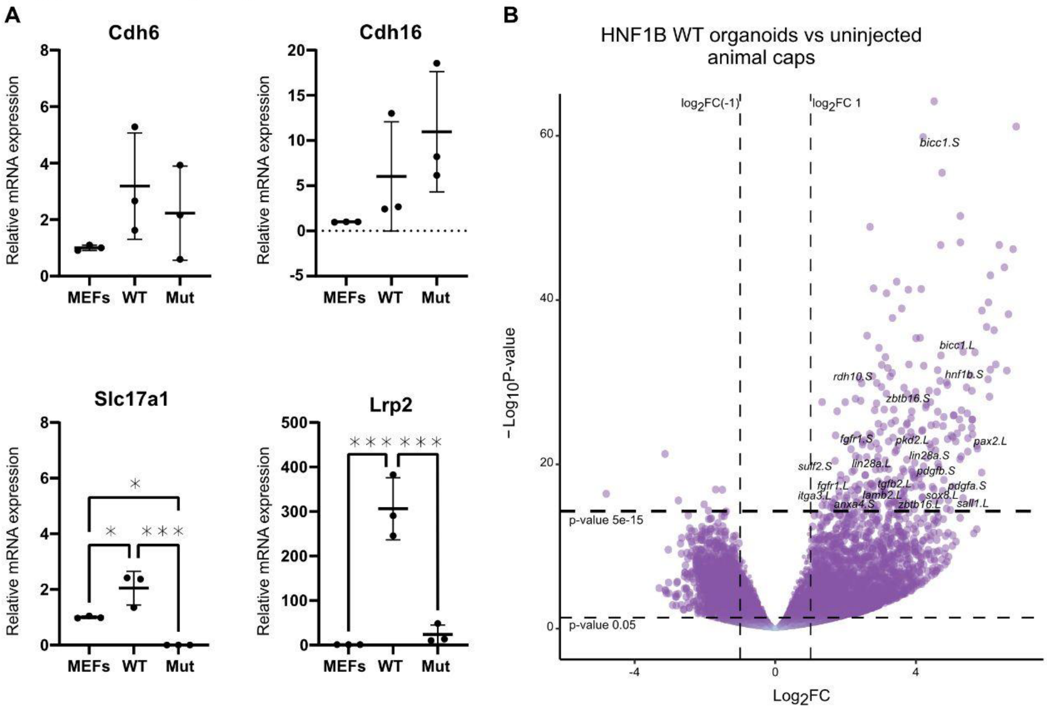

Supplementary Figure 4 related to Figure 4. **Transcriptional stability of the iRECs and genes essential for kidney development in *Xenopus* organoids**. **A**. Tubular differentiation markers *Cdh6*, *Cdh16*, *Slc17a1* and *Lrp2* in HNF1B WT and HNF1B R295C iRECs of later passages (passage 22) expression levels. Mean and standard deviation of n = 3 biological replicates; differences assessed by ANOVA with Tukey's correction for multiple comparisons, ***P < 0.001, *P < 0.05. **B**. Volcano plot of HNF1B WT vs animal cap tissue DEGs with highly confident (p-value 5e-15) genes essential for kidney development (GO:0001822 and GO:0072006). Cutoff lines for log_2foldchange are -1 and 1, for p-value 0.05.

Supplementary Figure 5

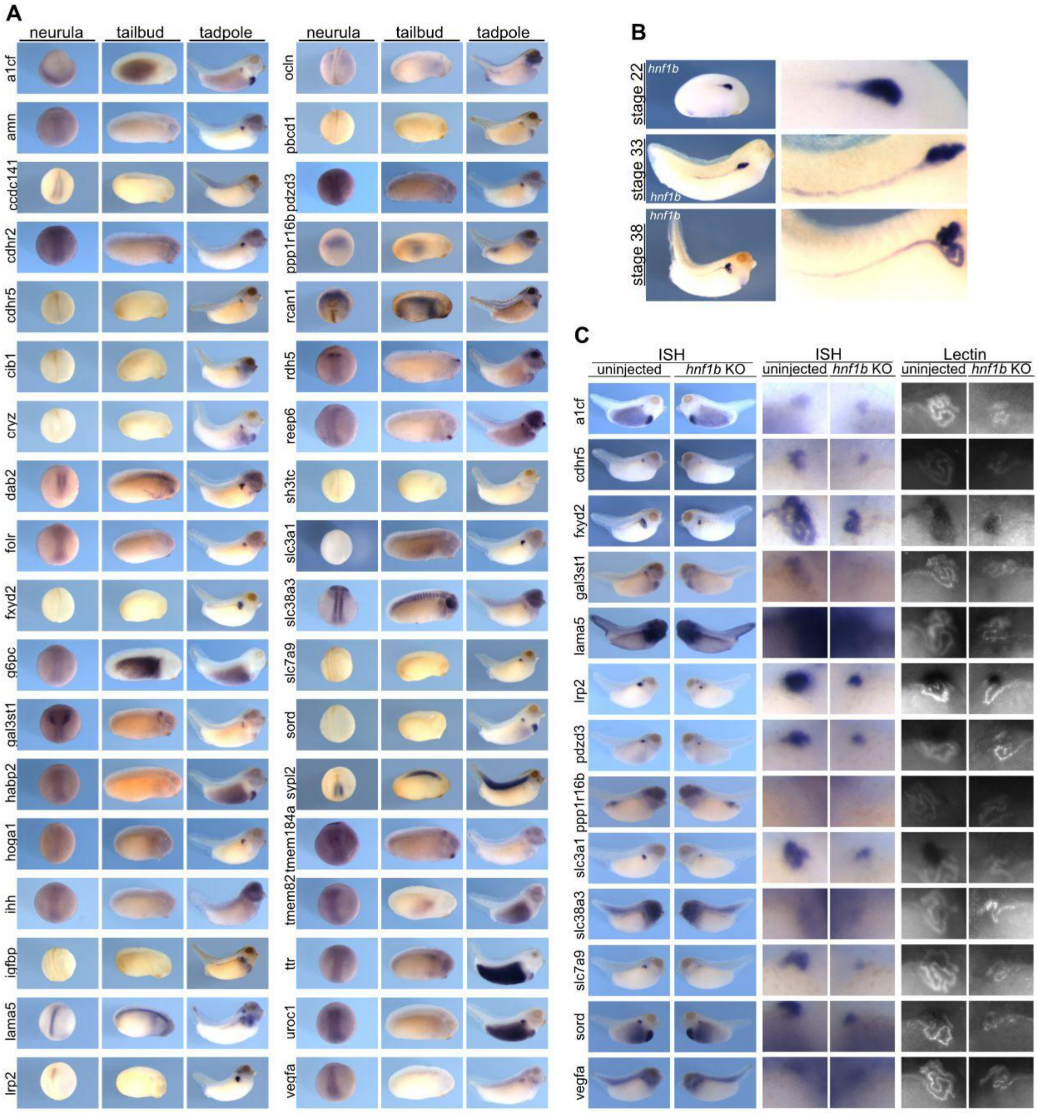

Supplementary Figure 5 related to Figure 5. ***In vivo* analysis of *hnf1b* and candidate genes**. **A**. Expression of the 36 candidate genes from the species overlap analysis in *Xenopus tropicalis*. *In situ* hybridization was conducted at three developmental stages - neurala, tailbud and tadpole. **B**. *Hnf1b* expression in *Xenopus laevis* at stages 22, 33 and 38. **C**. *In situ* hybridization combined with kidney specific LE-lectin stainings of 13 target genes in wild type and CRISPR-Cas9 *hnf1b* KO *Xenopus tropicalis* embryos.

Supplementary Figure 6

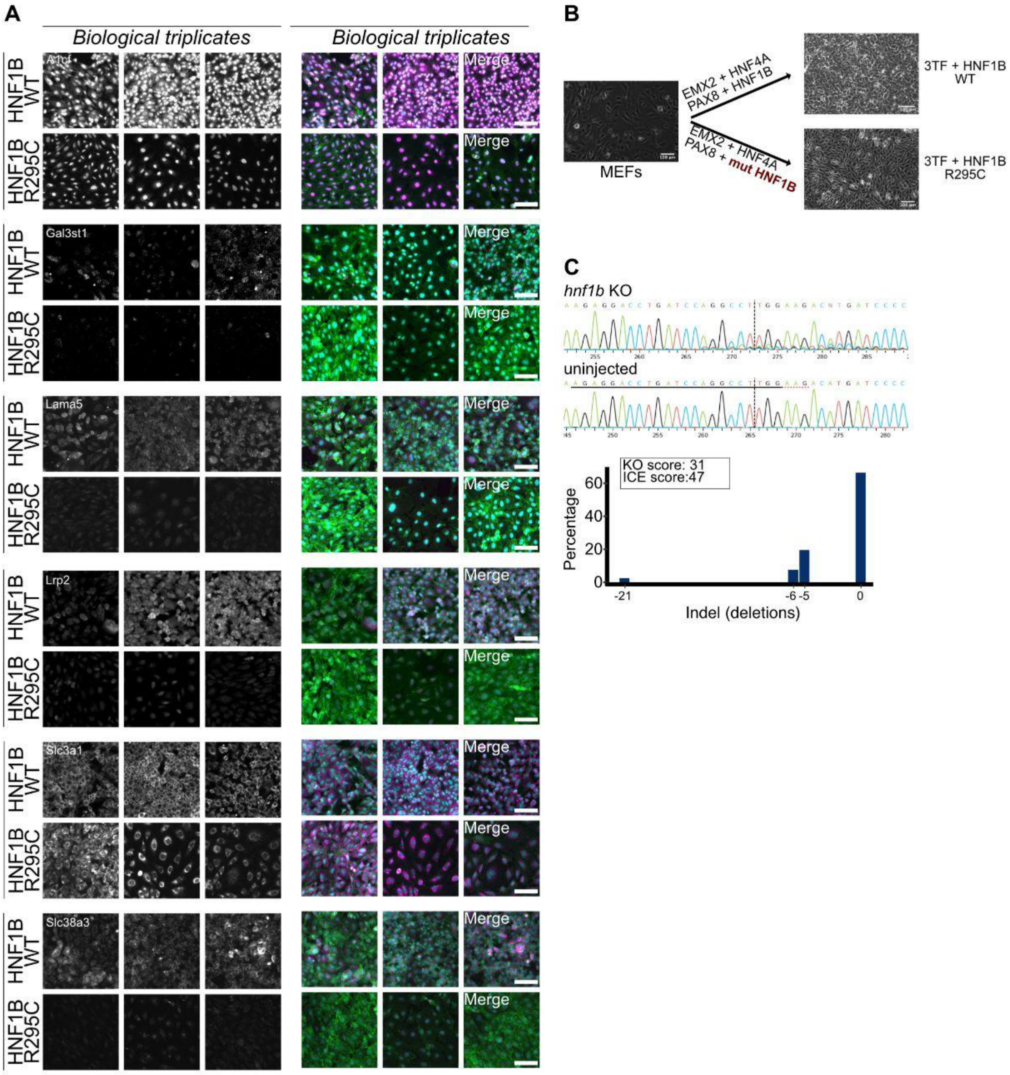

Supplementary Figure 6 related to Figure 6 and 7. **Analysis of HNF1B targets in iRECs and *in vivo***. **A**. Immunofluorescence stainings of three biological replicates of the reprogrammed iRECs (HNF1B WT and R295C) for six target genes. Merged images consist of iRECs cdh16-eGFP (green), target protein (magenta) and Hoechst (cyan) signals. **B**. Morphological changes of direct reprogramming using HNF1B WT (up) or HNF1B R295C (down) as seen in differential interference contrast images. **C**. Genomic sequence showing the *hnf1b* guide RNA genome targeted site (underlined) and sequencing chromatograms from KO and uninjected embryos. The bargraph represents the percentages of indels occurring at the hnf1b locus in the CRISPR/Cas9 KO experiments. KO and ICE scores based on three technical and five biological replicates.
